# Understanding Pathways Between Agriculture, Food Systems, and Nutrition: An Evidence and Gap Map of Research Tools, Metrics, and Methods in the Last 10 Years

**DOI:** 10.1093/advances/nmaa158

**Published:** 2021-01-04

**Authors:** Thalia M Sparling, Howard White, Samuel Boakye, Denny John, Suneetha Kadiyala

**Affiliations:** London School of Hygiene and Tropical Medicine, Bloomsbury, London, UK; Friedman School of Nutrition Science and Policy, Tufts University, Boston, MA, USA; Campbell Collaboration, New Delhi, India; International Center for Evaluation and Development (ICED), Nairobi, Kenya; Amrita Institute of Medical Sciences & Research Centre, Amrita Vishwa Vidyapeetham, India; London School of Hygiene and Tropical Medicine, Bloomsbury, London, UK

**Keywords:** food systems, nutrition, agriculture, metrics, methods, evidence synthesis, innovation

## Abstract

New tools, metrics, and methods in agriculture, food systems, and nutrition (A&N) research proliferated in the decade following the 2007–2008 food price crisis. We map these developments across themes derived from conceptual A&N pathways and expert consultations. We created an interactive Evidence and Gap Map (EGM) from a systematic search of published and gray literature since 2008, following Campbell Collaboration guidelines. We retrieved over 30,000 reports from published literature databases, and individually searched 20 online repositories. We systematically screened 24,359 reports by title and/or abstract, 1577 by full report, and included 904 eligible reports. The EGM consists of rows of thematic domains and columns of types of tools, metrics, and methods, as well as extensive coding applied as filters. Each cell of the map represents research surrounding a type of tool, metric, or method within a given theme. Reports in each cell are grouped by stage of development, which expand to a corresponding bibliography. Users can filter EGM reports by various characteristics. The 4 most populated domains were: diets, nutrition, and health; primary food production; water, sanitation, and hygiene; and environment and sustainability. The 4 most common types of metrics, methods, and tools were: diet metrics; footprint analysis (especially water); technology applications; and network or Bayesian analysis. Gaps represent areas of few or no reports of innovation between 2008 and 2018. There were gaps in reports and innovations related to: power or conflicts of interest; food environments; markets; private sector engagement; food loss and waste; conflict; study design and system-level tools, metrics, and methods. The EGM is a comprehensive tool to navigate advances in measurement in A&N research: to highlight trends and gaps, conduct further synthesis and development, and prioritize the agenda for future work. This narrative synthesis accompanies the EGM, which can be found at https://www.anh-academy.org/evidence-and-gap-map.

## Introduction

Agriculture, a primary source of food, income, and employment in low- and middle-income countries (LMICs), has received renewed focus in the last decade since the global food price crisis in 2007–2008 (1, 2). “Making agriculture work for nutrition”—nutrition-sensitive agriculture—climbed the international development agenda (3, 4). With the precipitous increase in diet-related chronic diseases and the threats of climate change to diets, sustainable food systems to optimize nutrition, health, and environmental outcomes has also gained momentum (5–7).

In the last decade, progress in this field has included several key developments. Research teams mapped agriculture or food systems and nutrition linkages, highlighting multiple direct and indirect complex pathways, which led to the development of new conceptual frameworks (5, 8–12). Researchers also set out to produce a more rigorous body of evidence using state-of-the-art methods linking agriculture, food systems, and nutrition (13, 14). Throughout these efforts, it became clear that there were inadequate tools, methods, and metrics to study the myriad of complex and dynamic relations between agriculture-food systems and nutrition outcomes (15–17).

Those working on agriculture, food systems, and nutrition (A&N) linkages began to develop, adapt, and use novel metrics, methods, and tools, often cutting across disciplines, to investigate these pathways. This also illuminated additional pathways between agriculture or food systems and nutrition outcomes, such as food environments, environmental factors, and food safety (18–22). Although the body of evidence on nutrition-sensitive agriculture has been recently systematically reviewed (23) and the state of food systems summarized (24), a systematic, inclusive portfolio of new methods and metrics, encompassing links between agriculture or food systems and nutrition has not. It is useful to take stock of these developments in order to support the production of effective and relevant research.

### Aims

The aim of this article is to identify, describe, and summarize innovation in tools, metrics, and methods that have been created and applied to understand A&N linkages since 2008 through a systematic mapping approach. To this end, we developed an Evidence and Gap Map (EGM) to describe advances in measurement. The result is an interactive map designed to facilitate access to a broad range of tools, methods, and metrics across the A&N research spectrum. The map and this synthesis highlight gaps and opportunities for future development, validation, and synthesis of tools, metrics, and methods. It can also be used to initiate collaboration and spur the interdisciplinary use of tools, metrics, and methods, and undertake prioritization within and across themes presented in the map. In turn, this can accelerate evidence-based actions to leverage agriculture and food systems for nutrition.

## Methods

We undertook a systematic mapping exercise, in the form of an EGM. Since there were no existing methods designed specifically for summarizing tools, metrics, and methods, we adapted approaches for effectiveness studies. A detailed methodological protocol is published elsewhere (25), a summary of which is provided here. The key features of the map are explained below and highlighted in **Box 1**.

**Box 1: Main features of the EGM**

- **Columns** of tools, metrics, and methods, by category
- **Rows** of thematic domains derived from conceptual frameworks
- **Bubbles** in each cell showing the number of reports, color coded by stage of development
- **Filters** (codes) that can be selected on and off, or in combination to show specific map characteristics
- **Units of measurement**, also available as filters, showing at what level a tool, metric, or method measures
- **Setting or geographic application**, also available as filters, showing where a tool, metric, or method was applied

An EGM is a comprehensive systematic synthesis and visual presentation of available evidence, or lack thereof (i.e. gaps), in fields of interest (26). For this EGM of tools, metrics, and methods, the first of its kind, we created 12 broad thematic domains (rows) informed by the prevailing A&N conceptual frameworks, pathways (8, 9, 16, 27–30), expert consultations, and extensive pilot-testing of the search strategy in order to ensure that the map is feasible and user-friendly (**Supplemental Figure 1**). **Table 1** includes the definition of food systems we used, and lists the 12 domains, with examples of the types of reports in each.

**TABLE 1:** Domains of influence on the agriculture or food systems to nutrition pathway

DOMAIN	EXAMPLES (for illustrative purposes only – not exhaustive)
**Food system definition** used: “all the elements (environment, people, inputs, processes, infrastructures, institutions, etc.) and activities that relate to the production, processing, distribution, preparation, and consumption of food, and the output of these activities, including socioeconomic and environmental outcomes” (31)	
Primary food production (growing,cultivating, raising, catching,harvesting, storing)	Agriculture, agroforestry, aquaculture, husbandry as a source of food; on-farm crop or food loss; yields; practices and techniques; harvesting; storage; processing for later consumption; seasonality; nutrient density/composition of crops; antinutrients at the production level
Value chains and food transformation	Food processing for retail; food processing for storage and later consumption; retail food distribution; nutrient additions or losses or preservation (nutrition-sensitive value chains); palatability; antinutrients (or absence/removal) at the food transformation level
Food safety	Aflatoxins; contamination; slaughterhouses; wet-market sanitation; foodborne disease; bulking steps; food preparation in households and other sites
Water, sanitation, and hygiene	Water footprint assessment, household water supply and water safety; distance to water; hygiene metrics; sanitation facilities; Water, Sanitation and Hygiene (WASH) checklists
Markets	Sale at markets; density; types; distance; accessibility; supply levels and availability; imports/exports; loss at market level
Economy	Purchasing power; consumption and expenditure; debt; economic resilience; income
Food environments	Food quality; food diversity, food availability, food accessibility (prices, distance to stores), determinants of food access/value, i.e. any work that falls under the definition provided by the CDC: “The physical presence of food that affects a person's diet; a person's proximity to food store locations; the distribution of food stores, food service, and any physical entity by which food may be obtained; or a connected system that allows access to food” (32)
	Food environments were earlier defined as “The collective physical, economic, policy and sociocultural surroundings, opportunities and conditions that influence people’s food and beverage choices and nutritional status.” (33)
Ecology, sustainability, and environment	Soil; forests; sustainability; climate change; resilience; water systems, agricultural water supply; water equity; biodiversity; land use
Policy and food governance, trade policy,and commitments to nutrition	Commitments to nutrition (private/industrial/government); food prices; systems research and development; structural investments; trade regulation; tariffs, taxes, incentives (e.g. subsidies); institutional capacity, function, and arrangements; decision-making processes
Conflict of interest	Conflicts of food corporations; conflicting investments; manufacturing or supply of nutritious or unhealthy foods and marketing practices
Food security	Food insecurity experiences of individuals, measurements of food shortages or volatility within households
Diet, nutrition, and health	Nutrition Knowledge, Attitudes and Practices (KAP), norms and behaviors, food consumption, nutritional status indicators (e.g. energy balance, micronutrient status, anthropometry); Non-Communicable Diseases (NCDs); food production-related labor burden, nutrition-related child illness; diet quality; bioavailability

The EGM columns represent the type of innovation in tool, metric, or method created and applied in A&N research. **Table 2** provides the definitions and categorization of tools, metrics, and methods with illustrative examples.

**TABLE 2:** Categories of tools, metrics, or methods used to study the agriculture or food systems to nutrition pathway

CATEGORY	EXAMPLES (for illustrative purposes only – not exhaustive)
TOOLS: a vehicle, technology, or an aid to collect information and data	
Technology measures/application	Geospatial applications: e.g. Geographic Infomation Systems, drones, spatial mapping
	Physical instruments, visual aids (e.g. wearable cameras, photovoice) or other measurement tools (e.g. accelerometers)
	Mobile/tablet-based and web-based applications, software, statistical programs: e.g. mobile data collection
	Biochemical tests (PCR, assays, LC, rapid diagnostics)
	Gene sequencing (18S, 16S, high-throughput, metabarcoding)
Research, survey, and interview tools	Quantitative tools: e.g. survey tools, new modules, new questionnaires
	Qualitative tools: e.g. new modules, new formats, new interview aids, new types of ethnography, focus groups, market surveys
METRICS: parameters (measures) or indices used for measurement, comparison, or tracking performance or outcomes of interest	
Measures and indices: continuous,including scales, dichotomousor polytomous	New types or versions of Likert scales Women Dietary Diversity Score, Months of Adequate Household Food Provisioning New classifications of growth measures, new body composition indices New dietary index
METHODS: the organization, process, or approach involved in a systematic inquiry of scientific data relations, generally referring to study design or the application of an analytical method to a topic	
Research design	Participatory design, surveillance systems, quasi-experimental methods, diagnostics, sampling
Analysis	Decision analysis, Bayesian theory, economic/cost analysis, optimization modeling, life tables, modeling studies, data transformation

### Search strategy

We employed a comprehensive published literature search of 2 databases, Web of Science and Commonwealth Agricultural Bureau (CAB) Abstracts using electronic screening with search terms (**Supplemental Methods 1**) (25). We also searched 20 organizational, project, and research databases for relevant gray literature, and performed backward-track citations in the bibliographies of key articles (**Supplemental Methods 2**). We searched for reports published from 1 January, 2008 to 31 December, 2018. We chose the 10-y period based on the renewed focus on and funding for A&N research that emerged following the global food price crisis in 2007–2008.

### Eligibility

The focus of this project was “innovation,” which was our most important criteria for inclusion of reports. For the purpose of this EGM, following extensive pilot testing and expert consultations (25), we adopted the following 3 criteria for innovation:

1. Completely new tools, metrics, or methods that were introduced after 2008 with no previous iterations.
2. Tools, metrics, or methods that existed prior to 2008 but that were significantly revised or modified since. As a “significant” change or modification can be subjective, we relied on the authors’ own assertions and explanations, and made an expert judgement collectively among the research team when unclear. For example, the Healthy Eating Index was developed in 1995, but was modified significantly after 2008. Therefore, we only include reports using versions published since 2008.
3. New or novel applications of existing tools and methods. This mostly entailed applying these across disciplines. When uncertain, we again relied on the authors’ description and justification, and secondarily made a collective decision among the author group. For example, Bayesian networks (BNs) were widely used prior to 2008, but their application to decision-making for agriculture and nutrition has popularized after 2008, and thus these applications were included.

Study types that demonstrated new innovations or novel applications could include a new study design, standard study designs using new or innovative tools, metrics, or methods, or studies specifically developing, piloting, or validating a new tool, metric, or method.

Reports were required to be written in English and had to explicitly describe a tool, metric, or method used for research. No geographic limitations were applied. Quantitative and qualitative research was included.

The majority of innovations are discrete in that they measure 1 link in the theoretical chain, but that link may not explicitly tie both ends together. For instance, dietary diversity metrics for women and children were improved for a number of reasons, especially as an outcome measure of nutrition-sensitive and agricultural interventions. The measure itself is not tied per se to agriculture. Therefore, we only included reports that were clearly related to agriculture and food systems ***and/or*** nutrition and nutrition-related health outcomes, but that were theoretically situated within 1 of the conceptual frameworks.

The prevailing conceptual frameworks include broad themes as underlying agriculture and food system determinants of human nutrition and health, such as soil health, land use, ecology, food environments, trade, food policy, and poverty. This mapping exercise thus reflects that breadth. Included reports could measure outcomes at any level: individual, household, crop, product or animal, farm or plot, community, district or subnational, national, or global.

### Exclusion criteria

We excluded reviews, in vivo plant and animal studies outside the context of the agriculture-food systems-nutrition pathway, animal feeding experiments, enhancement and therapeutic nutrition, and specific dietary supplement formulations, apart from routine population-based supplementation for women and children, and reports of niche or nongeneralizable populations. We excluded any reports of innovations identified or published after the search period concluded so as not to deviate from our published protocol (25) and systematic review guidelines (26), but these will be used as index reports to inform updates to the EGM. A detailed list of exclusion criteria is provided in **Supplemental Methods 3**.

### Screening and study selection

All screening and coding was conducted in EPPI Reviewer 4. For title and abstract screening, 2 independent researchers, under the supervision of TS and HW, screened the first 10%, with a third researcher providing a decision in the case of disagreement. Remaining items were screened by a single researcher, with 5% randomly checked by TS. Two independent researchers double screened all full-text articles included, and all disagreements were reconciled collectively by HW, TS, and SK.

### Data coding and analysis

A full coding classification and the numbers of reports with each filter code is available in **Supplemental Table 1**. All included reports were double coded by TS and 1 other researcher using a predefined data extraction form. All disagreements were reconciled by TS.

Each report was coded for a primary tool, metric, or method category. Some reports described multiple tools, metrics, or methods, or a composite of tools that made up an overall method. In these cases, we chose an overarching or “primary” tool, and listed any secondary tools, metrics, or methods in the data collection form.

Since several included tools, metrics, and methods cut across the thematic domains, each report was coded with ≤3 domains, therefore the numbers presented are not additive (i.e. the total number of reports is less than the number listed when adding all domains together). For instance, new analyses for crop water footprints were numerous, and these were coded as both “food production” and “water.” If they included specific metrics on sustainability and environmental aspects, they were also coded under “ecology, sustainability, and environment.”

We coded all items on the primary measurement unit, such as individual, household, crop, regional, or global. We also coded the geographic location or setting of application, as well as the stage of development or application of innovation (see below).

We used additional coding to indicate other characteristics of the tools, metrics, or methods such as gender, technology, children, microbiome, or economics. All codes can be selected from a list of filters in the map, which will then show only reports with that code. Some of the additional characteristics used as filters (like equity) are broad and are further identified into subcategories (such as gender, occupation, or socioeconomic status), which can also be selected. For example, all reports with a women's empowerment focus, such as those using the Women's Empowerment in Agriculture Index (WEAI), were first coded on their thematic domains, and also given a code for “gender” within the broader code of “equity,” as well as any other specific characteristics applicable. Finally, where several reports described the same or similar tool, metric, or method, these were given a code so that all reports using that innovation can be selected using a filter on the map. An example of this would be the Dietary Inflammatory Index code, which could be selected from a list of filters to show only reports using this index (Supplemental Table 1).

### Stage of development of tools, metrics, and methods

We assessed each item included (tool, method, or metric) for “stage of development” in place of a risk of bias assessment in effectiveness studies. We drew on literature on epidemiological indicator development as well as stages of innovation to create 4 ordinal categories (34, 35):

1. Concept development and pilot.
2. Feasibility or internal validity.
3. Demonstration and testing, external validity.
4. Adoption, generalizability, and widespread application.

For an expanded definition of these categories, see **Supplemental Methods 4**. Singular items that described a new tool, metric, or method as it was developed or piloted early on were coded as Stage 1. Items that were presented in a content validation or similar manner were coded as Stage 2. Items that showed evidence based on relations with external variables (criterion, convergent, or discriminant validity) or application to new settings were coded as Stage 3. Several items mentioning a tool, metric, or method developed or applied in a novel way after 2008 was evidence of “widespread” application, or Stage 4 of innovation.

We coded the stage of innovation from the cumulative evidence of adoption of the tool, metric, or method at the time of the review rather than the stage of development in each report at the time of publication.

Tools, metrics, and methods with >1 corresponding report can be explored in 2 ways: through a filter code in the EGM, and in **Supplemental Table 2** where all innovations are listed, with the number of reports noted for each. Tools, metrics, and methods with only 1 corresponding report can be found in “other” categories in the filters and listed in Supplementary Table 1.

## Results

We offer a narrative synthesis of our results in this article and have constructed an interactive EGM (see HTML file). The map visualizes the number of reports in a cell, segregated by stage of development, which expands to a corresponding bibliography. Gaps represent areas of few or no reports of innovation between 2008 and 2018. Users can filter the EGM reports by various characteristics. In this article, we also provide an analysis and lists (Supplemental Tables 1 and 2) of unique tools, metrics, and methods that have been identified through all included reports. In this synthesis, we offer illustrative examples to explain certain points; we urge readers to use the map to garner a more comprehensive view of tools, metrics, and methods advanced since 2008, and innovations that might be needed.

**FIGURE 1:**
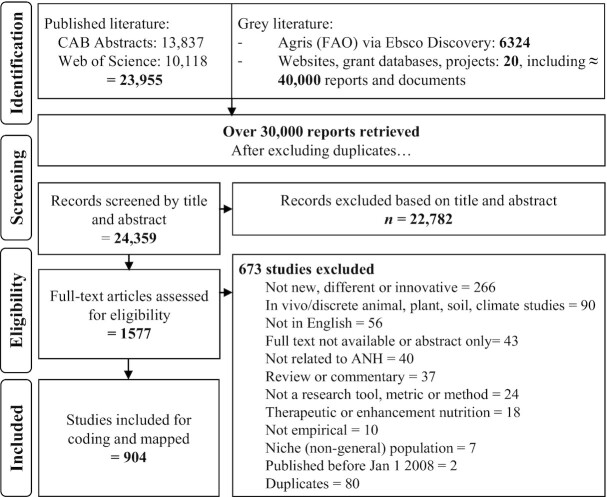
Flow chart of reports considered in the mapping. Each gray literature website or database required a unique search strategy. A description of website strategy can be found in Supplemental Methods 2. ANH, Agriculture, Nutrition and Health; CAB, Commonwealth Agricultural Bureau; FAO, Food and Agriculture Organization of the United Nations.

We retrieved 23,955 reports from CAB Abstracts and Web of Science. The gray literature search of 20 databases included 6324 documents from Agris and over 40,000 additional documents from United States Agency for International Development (USAID), World Bank, and the Consultative Group for International Agricultural Research (CGIAR) consortium databases. From the search hits in the gray literature, potentially relevant reports were imported into the main database for screening. After removing duplicates, we identified 24,359 items from both the published and gray literature searches for screening by title and abstract published in any language. We assessed 1577 full-text reports. Of those, 904 were eligible for inclusion and have been included in the map to date. The Preferred Reporting Items for Systematic Reviews and Meta-Analyses (PRISMA) diagram is presented in **Figure 1**.

## Types of Tools, Metrics, and Methods

**FIGURE 2:**
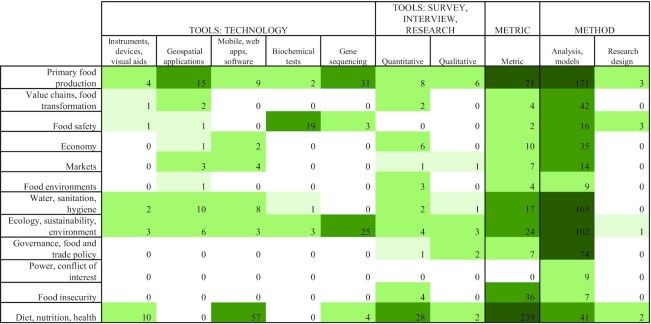
Heatmap of number of reports by thematic domain (rows), against innovations in types of tools, metrics, and methods (columns). Supplemental Table 2 is a list of the number of reports for each code, and **Supplemental Table 3** is a list of unique tools, metrics, and methods represented by reports.

The innovations that emerged from 904 reports (each assigned a single primary tool, metric, or method) ranged from new technology to new indices to the application of methods from other fields. The distribution of reports across thematic domains is shown as a simplified heatmap corresponding to the mapping framework in **Figure 2**. Columns will add up to 904 reports, but rows are not additive as there can be multiple domains coded for a single report.

The most common type of report described new metrics (354), followed by methods (330), especially new models and analysis techniques, and tools (220). As metrics and tools are mostly discrete and have fixed parameters, we were able to count both the number of reports and the unique tools and metrics that those reports represent. Many methods, especially models and analysis techniques, are applied in slightly different ways to a range of questions, therefore we identified similar methods, which we grouped, but may not be exactly the same. When clustering reports by the unique/similar tool, metric, or method, there were 182 new tools, followed by 128 new methods, and lastly 125 new metrics (Supplemental Table 2). The number of reports, as well as unique innovations represented in multiple reports is also shown in **Table 3**.

**TABLE 3:** Types of tools, metrics, and methods used to study agriculture, food systems, and nutrition pathways that are represented in the Evidence and Gap Map

Tool, metric, or method (TMM) type	Reports (based on primary TMM coding)	Unique TMMs
TOOLS	220	182
Technology measures and applications	164	128
Mobile apps, tablet, web, software	66	51
Gene sequencing	37	1^1^
Geospatial applications	23	38^2^
Biochemical tests	21	21
Instruments, devices, visual aids	17	17
Survey, instruments, and research tools	56	54
Quantitative tools	47	46
Qualitative tools	9	8
METRICS	354	125
METHODS	330	128
Analysis and models	323	121
Research design	7	7

1 We did not differentiate between types of genetic sequencing, but this includes 16S and 18S pyrosequencing, metabarcoding, and others.

2 23 reports were identified where the primary tool was a geospatial application, however, there were 38 reports where a geospatial application was part of the TMM but not the primary component.

Reports highlighting new metrics were mostly in the domains of diets, food production and food security, and ecology, sustainability, and environment. Scores, scales, and indices were introduced, with and without validation, for both general purposes and within specific study settings. Metrics with the most numerous reports were the new WHO Infant and Young Child Feeding Indicators (93 reports), the Healthy Eating Index (38 reports), and the WEAI (36 reports), which each have a filter code in the map.

The biggest groups of methods were specific analytical approaches and models, especially water footprint analysis using the Water Footprint Network's methods (36), Life Cycle Impact Analysis, Agricultural Sector Risk Assessments, and Land Governance Assessment Frameworks (a count of those mentioned can be found in Supplemental Table 2, and as filters on the EGM). New models and algorithms on complex systems were advanced, especially the novel application of BN analysis (by far the most prevalent), probabilistic models, Artificial Neural Networks (ANN), systems theory, path analysis, stochastic modeling, and others. These modeling methods were used across the board to describe and quantify complexity in the agricultural, food systems, or nutrition space. Some of these modeling methods and tools were used in the most traditional statistical sense, and some of the models were used as qualitative or mixed-method decision and consensus tools with multistakeholder partnerships or members of a community. There were 62 reports describing new decision support tools, which is available as a filter code in the interactive map (also listed in Supplemental Table 1). There were only 7 new research designs identified.

The most innovation in tools was in technology measures and applications. New mobile applications, software, or programs comprised the largest group of technology tools. Genetic sequencing, also called metabarcoding, gained momentum as a new method in the agriculture-nutrition space. Sequencing techniques were used to improve rigor in studies of diversity, whether in water, soil, livestock, or fisheries. Geospatial applications were common, both as a primary and secondary tool. There were a group of reports that described advances in biochemical assessments for food safety, particularly ones that developed new tests (assays, rapid diagnostics, and chromatography) to detect antibiotic residues in water and food. New instruments, devices, and visual aids were represented in many reports, but less than the other categories. Of the 54 reports describing new survey and research tools, only 9 of these were qualitative (Table 3). Each of these groups can be filtered and further explored in the map.

### Thematic domains

**FIGURE 3:**
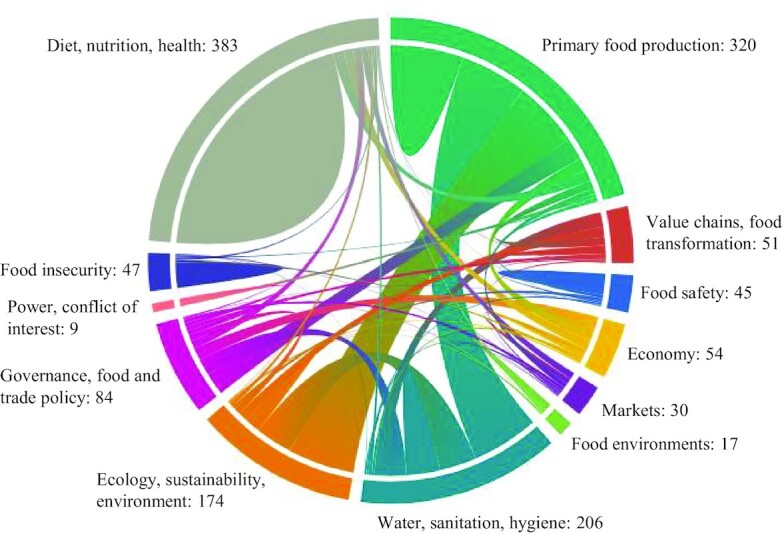
Chord diagram of thematic domains within agriculture, food systems, and nutrition pathways: total reports coded on each domain (listed on the periphery), the number of reports with only a single domain are unconnected to others in mounds, and reports linking to a second domain are shown in chords sized proportionally to the number of reports connecting the 2 domains.

**Figure 3** shows the total number of reports coded on each domain on the periphery of the circle. The unconnected “mounds” show the proportion of reports coded only on that single domain. For instance, many dietary metrics are discrete measures, and therefore are unconnected to other domains. Chords represent the number of reports that are coded on 2 connected domains, such as crop water footprint assessments (WFAs), whose characteristics are inherently about “water” as well as “primary food production.” A decision tool for policymaking on food safety risks would contribute to the chord connecting “food safety” and “governance, food, and trade policy.” The most common thematic domain by number of reports was diets, nutrition, and health, followed by: primary food production; water, sanitation, and hygiene; and ecology, sustainability, and environment. Further visualization can be seen in the EGM.

#### Diets, nutrition, and health

We identified a plethora of diet and nutrition tools, metrics, and methods, especially healthy diets (95 reports), food insecurity and dietary diversity (84 reports), and micronutrients (41 reports), among others. There were many reports describing new metrics and methods specifically for children and adolescents, especially to measure their diets. The WHO published new standard versions of Infant and Young Child Feeding (IYCF) indicators in 2008 (37), so reports using these were included. Advances were also made in web-based programs, mobile applications, software, and nutrition modeling. Although there were the most reports in the thematic domain of diet, nutrition, and health, there were fewer unique tools because many reports describe the same metric or tool: 66 individual new dietary metrics were identified within the 383 reports in this domain. Within this subdomain, there are many filters available in the interactive EGM for groups and individual tools, metrics, and methods (such as the Optifood tool). The number of reports on each of these aspects can also be found in Supplemental Table 2.

There were almost no new tools, metrics, or methods designed for impact-level nutrition and development assessment such as anthropometry or biomarkers of nutrients—most new tools and metrics were diet related. Very few or no new tools, metrics, and methods were developed for nutrition knowledge, attitudes, and practices, other diet-related health outcomes, social nutrition, and sociocultural aspects of diets and health, or eating behaviors.

#### Primary food production

Primary food production was the second largest domain by number of reports (Figure 3). New methods for considering crops and yields was mostly made up of WFAs. One hundred and eighteen reports were categorized under both the primary food production and water domains. Soil quality indicators and assessment methodologies were also common, especially due to the increasing use of genetic sequencing to determine the microbiome of soil under different practices or land use. For example, of the 34 reports assessing the microbiome of various things (gut, feces, aquaculture ponds, breastmilk, etc.), 23 of them were focused on soil.

Animal husbandry and aquaculture were themes with many new tools, metrics, and methods. The WEAI, was widely adopted in the last decade, as shown by 36 reports employing it from all over the world (available as a filter in the map within “Equity” and in Supplemental Table 2). The WEAI was also coded as “primary food production” because it specifically measures empowerment of women engaged in agricultural livelihoods. Land use metrics, including land governance assessment and climate-smart agriculture frameworks were newly developed and adopted, primarily by the World Bank.

There were few new tools that emerged to study yields, nutritional value of crops, or postharvest loss, farm-level vulnerability, or agroforestry, as examples. There were no new tools, metrics, or methods that we identified to measure crop yields in households or small-holder farms. Nutritional functional diversity was used in several ways—at a national scale, as well as linking family farms to micronutrient status of individuals, which revealed interesting relations, but it was not widely adopted (38).

#### Water, sanitation, and hygiene

WFA methodologies represented by far the most innovation and uptake, which were applied to individual crops, water points, supply chains, cities, districts, nations, and global trade. Life cycle impact assessments were also redesigned to capture multifaceted aspects (especially environment, sustainability, cost, and benefit/utility) of single crops, systems, or value chains, in which often were nested WFAs. There were many reports of new discrete metrics of water insecurity and stress, which were often included as part of a WFA.

There were no tools, metrics, or methods on sanitation or hygiene, except for the development of several household-level water insecurity scales or indices. Water insecurity indices were developed as an accompaniment to food insecurity indices and are being widely adopted. Some new methods and metrics for irrigation and water quality emerged.

#### Other thematic domains

“Ecology, sustainability, and the environment” was a thematic domain of 174 of the new tools, methods and metrics. Some of these were because water footprints included explicit ecological aspects (65 reports were coded also with the water domain), but there were also innovations in how to measure aspects of climate change, land use, biodiversity, and others in relation to agriculture-nutrition pathways. Of the reports falling under the theme of ecology, sustainability, and the environment, there were 117 reports that shared the primary food production domain.

There were 84 reports on governance, food, and trade policy (mostly as a secondary domain code), but many gaps within this category still exist, especially systems- and macro-level tools, metrics, or methods. This is also the case with food transformation and value chains, which are also related to markets and trade. Almost no tools and metrics explicitly for nutrition value chains were identified, although 1 conceptual framework was identified (39). Food security as a domain had 47 reports, which accounted for 10 new metrics. This is most likely due to widely accepted global indices such as the Food Insecurity Experiences Scale (FIES) and the Household Hunger Score (HHS) (validated and published after 1 January, 2008), and the Household Food Insecurity and Access Scale (HFIAS), which was not included in the map as it did not qualify as “new.”

Food environments, markets, and food safety domains had relatively few reports of new tools, metrics, or methods. New tools, metrics, and methods for analyzing food environments were mostly used in high-income countries. However, the Nutrition Environment Measures Survey short version (NEMS-S) was adapted and validated in Brazil (40), and spatial-temporal BNs and geospatial analysis were adapted to measure food environments in LMICs (41–45). Just a few new indices and tools were designed for markets and economy, such as the Cost of Diet tool (46, 47), the Cost of Dietary Diversity, and the Cost of Nutrient Adequacy indices (48), which were also included in the diet and nutrition domain.

There were 9 reports relating to power or conflict of interest in the food system as a thematic domain. These reports described a single method, the Land Governance Assessment Framework, which includes a specific focus on trade-offs and assessment of power dynamics.

### Measurement units

**FIGURE 4:**
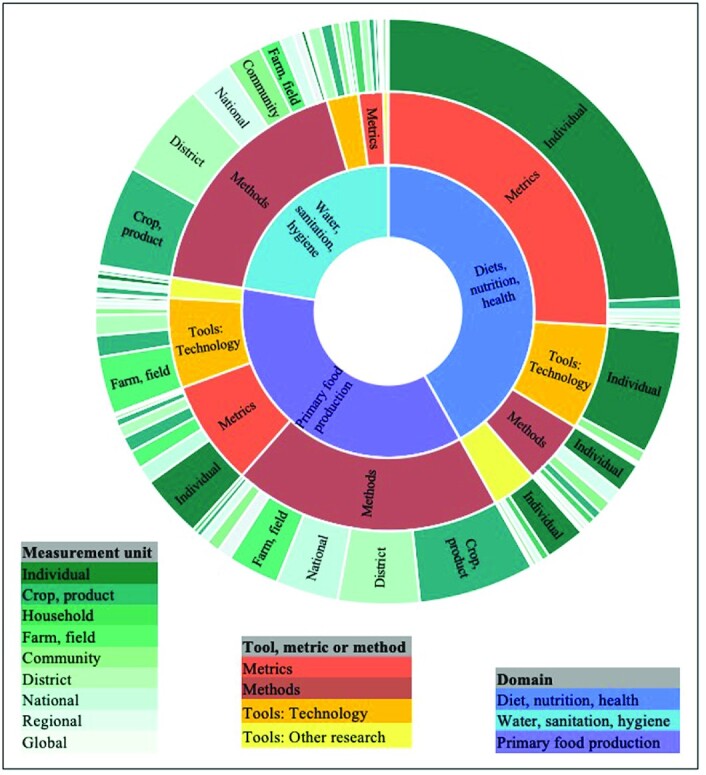
Proportion of reports on agriculture, food systems, and nutrition tools, metrics, and methods (middle ring) by measurement unit (outer ring) for the 3 largest thematic domains (inner ring).

In **Figure 4**, the measurement unit of reports (outer ring) is shown proportional to the type of tool, metric, or method (middle ring) for the 3 largest domains (inner ring: diets, nutrition, and health, primary food production, and water, sanitation, and hygiene). Tools, metrics, and methods were designed to use data collected at every level, ranging from a human individual, to crops, products, units of water, animals, factories, schools, river basins, geographic areas, entire nations, and global networks. For instance, in the section on water, sanitation, and hygiene, the majority of innovation is in methods, which are largely measured at the crop or district level. Far more underrepresented are those used for midlevel and macro measurement, or tools, metrics, or methods for measuring system-level interactions, such as those in communities, nations as a whole, and across global networks. Some tools, metrics, and methods have predefined units of measurement in order to be viable. For instance, most dietary metrics can only be used with individual dietary data. Other tools, metrics, and methods are more flexible in the data inputs that can be used. Some methods, such as the “footprint family” require various calculations across crops, landscapes, and individuals.

### Setting/geographic application and stage of development

**FIGURE 5:**
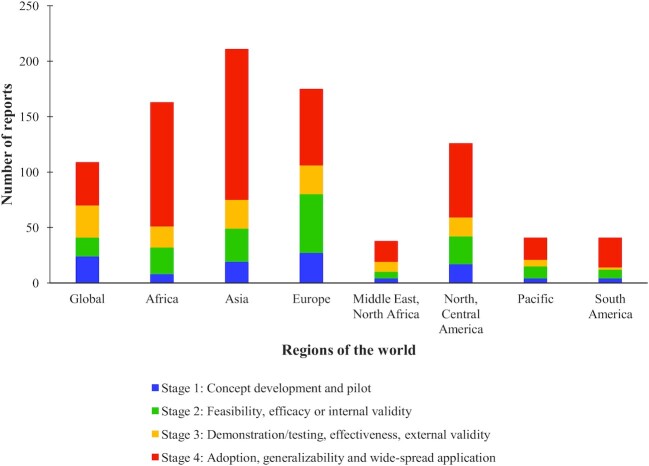
Number of reports describing new tools, metrics, and methods for studying agriculture, food systems, and nutrition by region, stacked by stage of development.

**Figure 5** shows the number of reports, stacked by stage of development, which can also be applied as filters in the EGM. Reports came from all over the world, but were dominated by China, European nations, Africa, and the USA. The Middle East and North Africa did not host as much innovation, and the Pacific region was almost entirely dominated by Australia and New Zealand, with few innovations emerging from or being applied to the Pacific Island nations. Very few tools, metrics, or methods were applied in Central America or the Caribbean, and in South America, the most innovation came from Brazil and Argentina. The geographic spread of reports was characterized by searching only English language databases, which is further discussed below. The majority of reports were in Stage 4, although this might be affected by publication bias, since those in widespread use will be published more often.

### Crosscutting characteristics (filters in the interactive map)

**FIGURE 6:**
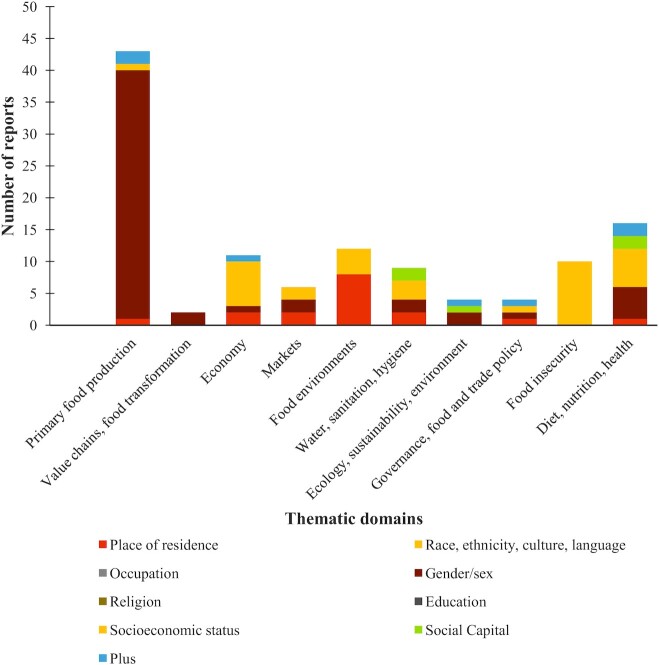
Number of reports demonstrating aspects of equity by thematic domain in the agriculture, foods systems, and nutrition pathways.

**Figure 6** shows the number of reports on equity, stacked by type of equity, across the thematic domains. Characteristics common to reports, or representing certain themes in the literature beyond or within the main framework (available as filters in the map) showed both proliferation and gaps in certain areas, as illustrated in **Table 4**. Tools, metrics, and methods specific to children (165 reports) and aspects of technology (160 reports) were the most common characteristics. Although there were many reports coded for the equity subdomain, the majority related to gender (just 1 aspect of equity) and many were applications of the WEAI. Other aspects of equity such as religion, occupation, age, and others were not well represented in reports.

**TABLE 4:** Summary of reports with crosscutting filters

Crosscutting filter	Count
Children	165
Technology	160
Equity (PROGRESS+)	81
Economics, poverty	53
Microbiome	34
Carbon/energy	30
Shocks and humanitarian context	26
Disabilities and ill-health	26
Food loss/waste	7
Private sector engagement	2

The map revealed clear areas where there were few or no innovations in reports describing private sector engagement, food loss and waste, shocks and the humanitarian context, and disabilities. There were reports related to economics, poverty, and inequality. Some assessed cost-effectiveness of nutrition-sensitive programs in novel ways, 1 through using the Cost of the Diet tool applied to fortification strategies (47), and 1 using a BN framework to determine agricultural project cost (49). However, novel direct measures and methods related to poverty and inequality were few.

### Strengths, limitations, and interpretation

This is the first and the most comprehensive systematic review to date, and the first EGM, to summarize tools, metrics, or methods in A&N research. This EGM has many strengths. We employed a rigorous and thorough systematic search, screening, and coding process to minimize misclassification. We were informed by consultation with subject matter and methods experts throughout the process. There is a vast breadth of subject matter included, which can be valuable to researchers across many disciplines. Reports in the EGM are able to be filtered by topic, setting, or geographic location, type of tool, metric, or method, stage of development, and other key thematic categories. We offer a summary and analysis of both the individual reports, and the unique innovations included in the map, as well as areas where there are few or no innovations identified. The unique interactive map as a tool allows researchers to explore work and identify gaps within their domains of expertise, utilize existing methods and measures from their own and other disciplines, compare aspects and characteristics of tools, metrics, and methods, and prioritize areas for future work.

Achieving breadth and depth of the map while maintaining a rigorous and systematic process meant that searching additional databases, especially non-English language databases, was not feasible given the time, funding, and language capabilities of the research team. Optimizing the search strategy for this wide scope was challenging; yet, we still screened over 30,000 reports from 2 databases alone. Innovations from regions where English is not the official language could be underrepresented, although some English-speaking regions still have less corresponding reports, such as the Pacific. We aired on the side of inclusion and breadth, and although we followed systematic procedures, have likely reduced bias, but may not have completely eliminated it.

We summarized the number of reports that describe new or new applications of tools, metrics, and methods in the A&N space. Some reports described multiple innovative components that fit distinctly within different types of tools, metrics, or methods. Therefore, we chose the “primary” tool, metric, or method to code the item, and listed the others that were secondary. This may have resulted in certain pragmatic groupings that could be debated. Similarly, the thematic domains for the EGM were distilled from conceptual frameworks and expert consultation, but there are alternate ways of grouping these. We worked through several iterations of defining these, so that the EGM would function as a whole.

Tools, metrics, and methods with no previous iterations were straightforward to classify. However, as certain tools, metrics, and methods evolved, defining what constituted “significant” evolution was not always straightforward. Metrics are discrete and have a standard construction, and are therefore easier to identify and evaluate. New methods are harder to identify and classify, especially when attempting to discern whether a combination of older metrics and methods, or applications to new fields and subtopics, is, in fact, new or novel. In the case of uncertainty, we relied on the authors’ explanations and background or discussion sections to frame the work, conducted Google Scholar searches to establish a timeline of evolution, consulted with experts in each of the domains, and took collective decisions within the research team when necessary. This process also helped us determine the stage of development of tools, metrics, and methods.

Well-populated categories and cells on the EGM might mean that these categories are dominated by certain types of innovations, not necessarily that there are no gaps. Cells with few or no reports in them could indicate that there might be well-developed older methods, metrics, and tools to measure intended relations. Although tools, methods, or metrics introduced prior to 2008 might have been widely used (such as child length or crop yields), there could still be scope for innovation if the area of study is deemed important and there is a scientific basis for innovation. Regional gaps may also be filled by searching non-English language repositories.

We recognize that highlighting innovation could lead to overlooking these well-developed, older methods; or introduce tools, metrics, and methods that are cumbersome, complex, or overly technical for practical use. Conventional risk of bias tools are not applicable or appropriate to gauge these issues related to “quality” of tools, metrics, or methods (such as their inherent value or lack thereof, appropriate use, whether they are overly technical for practical use, etc.). Interpreting the nature and value of further innovations is beyond the scope of the review. We therefore limit our interpretation of gaps to mean few or no reports of innovation between 2008 and 2018.

Even given these limitations, the investment in measurement of complex A&N linkages came from clear evidence that existing tools, metrics, and methods were unable to capture the complexities of these pathways (12), and thus we map advances with the intent to examine progress against this articulated need. When interpreting the EGM and its results, it is important not to prioritize topics and themes only based on the number of reports in any given category, but to delve into the diversity of tools, metrics, and methods within each category.

Lastly, this EGM is truly a map: it is a tool for navigation, showing advances since 2008 and the extent of their use. It will not definitively order or prioritize either advances or gaps given the breadth of themes, disciplines, and applications of tools, metrics, and methods. It is designed, however, to catalyze and facilitate efforts by stakeholders to prioritize investment in A&N research based on their vantage points and domain expertise, and also when diverse perspectives and consensus are needed. An agronomist working on more nutritious varietals might want to also consider aspects of water and ecology. Groups interested in value chains and market dynamics might want to consider gender aspects more thoroughly. Donors may use the map to inform their strategic plans and opportunities for funding.

There may be gaps that are more pressing to fill for various reasons. For example, threats to supply chains and the increasing interconnectedness and globalization of the food system might mean that system-level tools, and those that measure the widest “arcs” of trade and economy in food will be paramount. Food safety and food environment measures have been improved as a direct response to the very real risks of rapidly changing culture and consumption, and this will continue. The increasing number of conflicts that exist at any 1 time, both acute and protracted, might mean that tools and methods to accurately measure nutrition and health impacts in these contexts might be prioritized over others. The growing concern over links between climate change and agriculture or diets could signal the importance of investing in tools, metrics, and methods to study these aspects better. The proliferation of healthy diet and sustainable diet metrics already indicates some of these changing priorities. Ultimately, however, the user will have to examine the features and contents of the EGM based on their domains of interest and expertise to advance the field meaningfully.

## Conclusions

Clear trends emerged in measuring pathways between agriculture, food systems, and nutrition. There were many innovations combining measurement across domains, such as mixing and matching from water, food production, ecology, nutrition, health, and others to capture complexity and new levels of impact. There were “popular” new approaches, including new dietary metrics, food production methods, WFAs, gene sequencing, BNs, and system dynamics models. These may reflect emerging priorities to address sustainability and climate change and improve health through diets, and emerging capacities in biochemistry, computing power, rapid diagnostics, complex modeling. There were also clear gaps where no new tools, metrics, or methods either exist or have changed or been newly applied in the last decade (**Box 2**). These included innovations in ways to measure power and conflicts of interest, food environments, governance, investment, fluid or fragile states, markets, and economy. Many more niche gaps, even in the most populated domains, were also observed (**Box 3**). Although it is beyond the scope of this EGM to definitively or prescriptively prioritize the gaps noted, it is conceivable that as policymakers and funders undertake their risk analyses of, planning for, and response to the most serious food and nutrition threats, stakeholders will implicitly or explicitly rank gaps. If food system shocks occur, methods of capturing real-time data and learning from “natural experiments,” such as through surveillance systems, might evolve quickly. If global markets shift dramatically, the need to measure inequity and address vulnerability will be crucial.

**Box 2: Key gaps in type of innovation**

- Qualitative methods
- System-level tools, metrics, and methods
- Research design
- Dynamic, surveillance, ongoing and real-time research innovation
- Coproduction, participatory research
- Instruments and devices

**Box 3: Key thematic gaps**

- Power and conflict of interest in the food system, food industry, corporate engagement
- Food environments, food choice, and eating behavior
- Food loss and waste
- Short-term shocks, humanitarian contexts, emergencies
- Long-term vulnerability, migration, fragile states
- Food systems trade, trade-offs, and governance
- Markets and value chains for nutrition and health outcomes
- Agriculture and nutrition knowledge, attitudes and practices, norms and values
- Equity and inclusion, especially types other than gender

We imagine that the EGM will help to define key questions that remain about studying complex agriculture to nutrition pathways. It can be used as a resource to investigate developments within certain domains, types of application, or within specific research parameters. In which areas are existing methods sufficient and therefore should be the focus of research investment that does not require development of new tools, metrics, or methods? What questions remain that cannot be answered through methods and metrics that exist, and therefore should be the focus of research innovation and nontraditional investigation? This synthesis project highlights which thematic domains have been left behind in the development of new tools, metrics, and methods in the last 10 y, and what tools, metrics, and methods have been developed or applied that can be developed further and brought into widespread adoption.

One possible next step of the research is to conduct systematic reviews of specific tools, methods, or metrics where there are substantial measurement advances, with a view to producing practice-based guidelines. We will undertake stakeholder consultations to decide on expanding and updating the map, which could include automating the search strategy and further coalescing reports around their respective tools, metrics, and methods. Overall, the EGM is a navigation tool and a resource for priority setting. It can be used to avoid duplication of work, promote crossdisciplinary concepts, applications, and partnerships, and drive evidence-based investments in future agriculture, food systems, and nutrition research.

## Supplementary Material

nmaa158_Supplemental_File

## Notes

This work was funded through the Innovative Methods and Metrics for Agriculture and Nutrition Actions (IMMANA) program; which is funded with UK aid from the UK government (UKAid) and the Bill and Melinda Gates Foundation (BMGF). UKAid and BMGF had no role in the design, analysis, or writing of this article. The content of this publication is solely the responsibility of the authors.

Author disclosures: The authors report no conflicts of interest.

Supplemental Figure 1, Supplemental Methods 1, 2, 3, and 4, and Supplemental Tables 1 and 2 are available from the “Supplementary data” link in the online posting of the article and from the same link in the online table of contents at http://academic.oup.com/advances/.

Abbreviations used: A&N, agriculture, food systems, and nutrition; BN, Bayesian network; EGM, Evidence and Gap Map; LMIC, low- and middle-income country, NEMS-S, Nutrition Environment Measures Survey short version; WEAI, Women's Empowerment in Agriculture Index; WFA, water footprint assessment.
