# Supplementary material for: Understanding Pathways Between Agriculture, Food Systems, and Nutrition: An Evidence and Gap Map of Research Tools, Metrics, and Methods in the Last 10 Years
Source: Adv Nutr. 2021 Jan 4;12(4):1122–36. doi: 10.1093/advances/nmaa158 (PMC8321871; doi:10.1093/advances/nmaa158)
Supplement: nmaa158_Supplemental_File [file nmaa158_supplemental_file.docx]

Understanding pathways between agriculture, food systems and nutrition: An evidence and gap map of research tools, metrics and methods in the last ten years.

*Sparling, T., et al.*

**ONLINE SUPPLEMENTARY MATERIAL**

**Supplemental figure 1: Conceptual frameworks of agriculture or food systems pathways to nutrition**

**Figure S1-Framework 1:** Kadiyala S, Harris J, Headey D, Yosef S, Gillespie S. Agriculture and nutrition in India: mapping evidence to pathways. Annals of the New York Academy of Sciences, Volume: 1331, Issue: 1, Pages: 43-56, First published: 05 August 2014, DOI: (10.1111/nyas.12477)


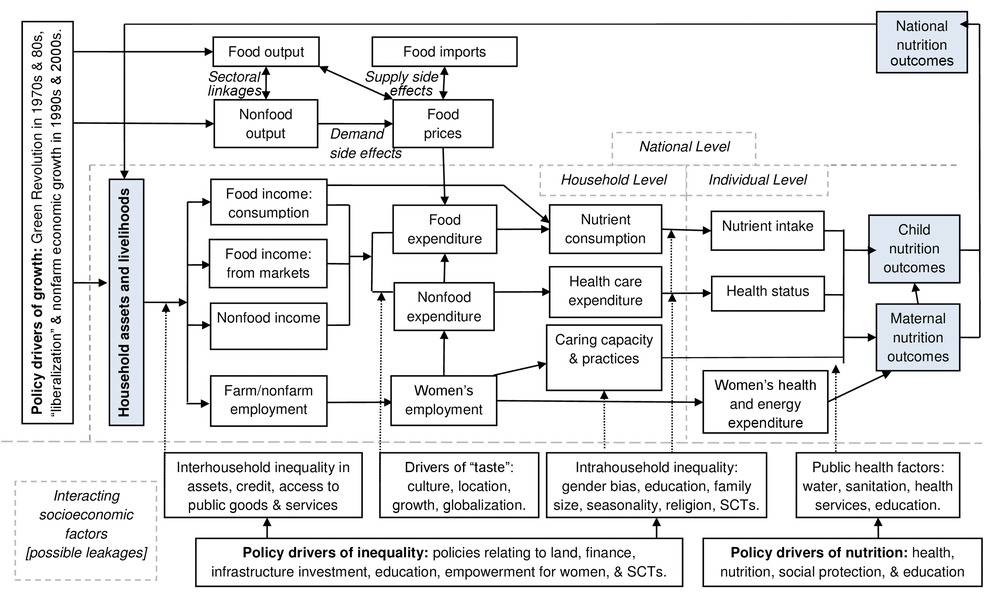


**Figure S1-Framework 2:** Hawkes C, Turner R, Waage J. **Current and planned research on agriculture for improved nutrition: A mapping and a gap analysis.** A report for DFiD: Leverhulme Centre for Integrative Research on Agriculture and Health (LCIRAH), Centre for Sustainable International Development, University of Aberdeen; 2012. Reprinted with permission.


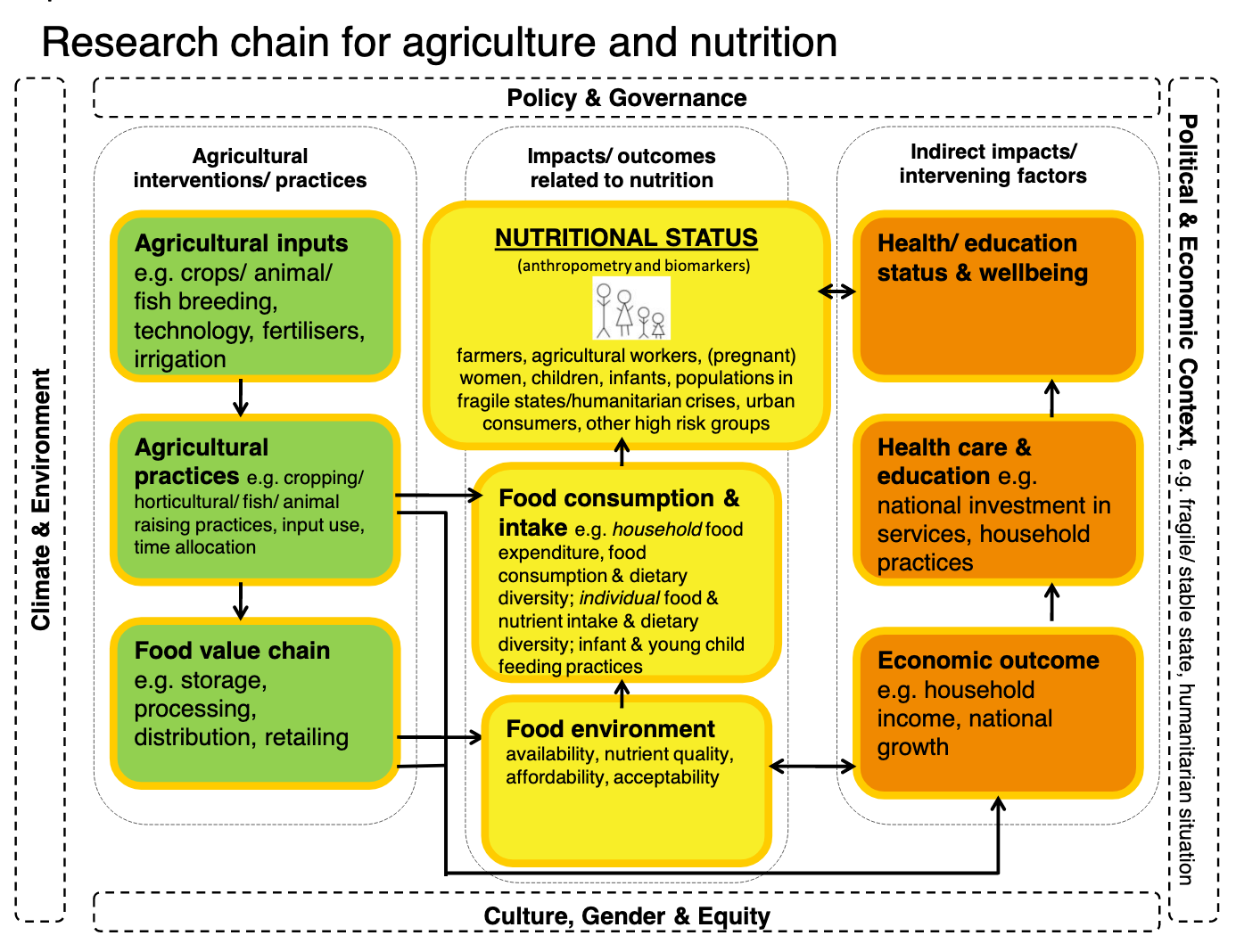


**Figure S1-Framework 3:** Herforth A, Nicolò GF, Veillerette B, Dufour C. **Compendium of indicators for nutrition-sensitive agriculture**. Rome, Italy: Food and Agriculture Organization of the United Nations; 2016. Reprinted with permission under the Creative Commons Attribution License.


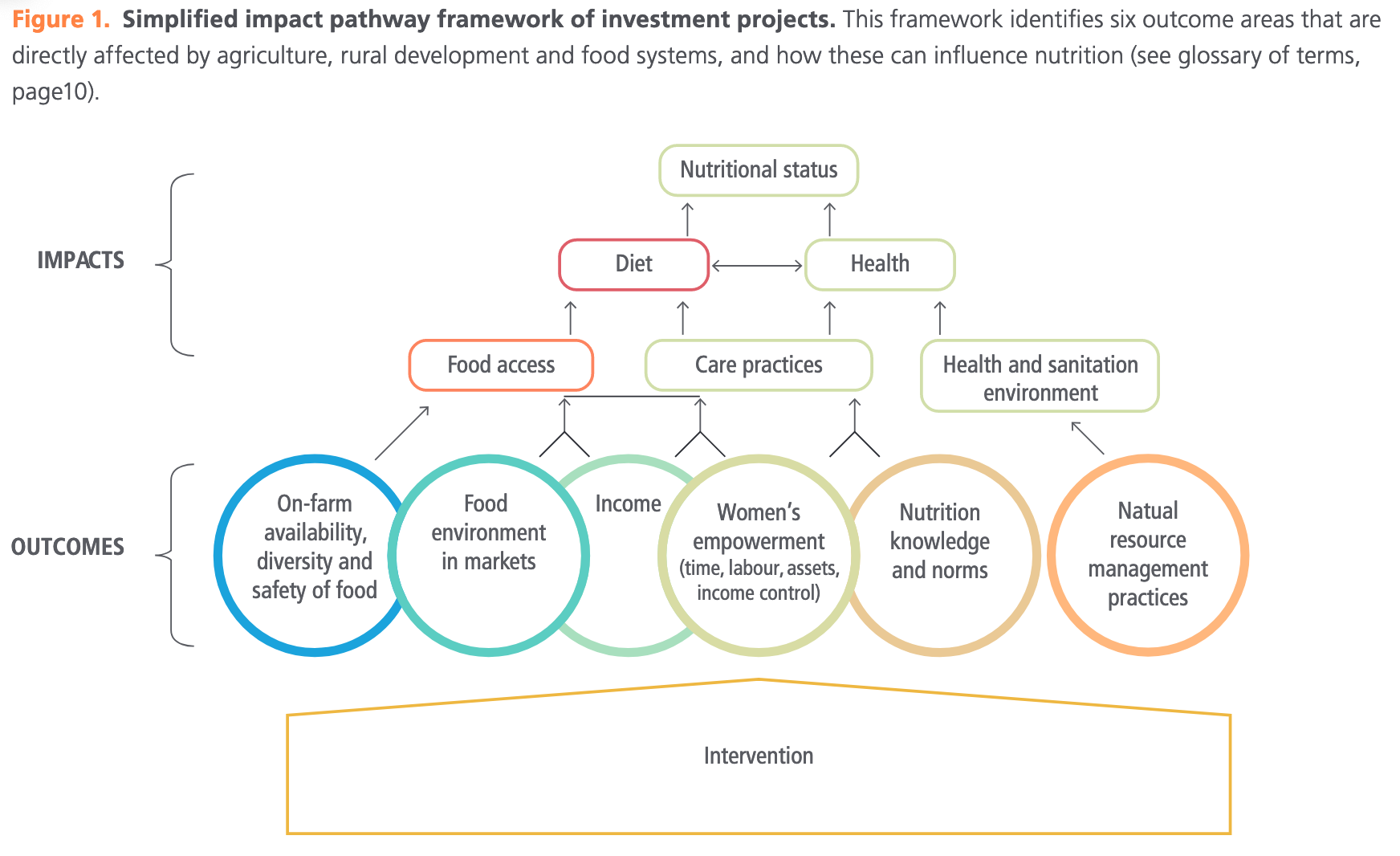


**Figure S1-Framework 4:** Tuomisto HL, Scheelbeek PFD, Chalabi Z et al. **Effects of environmental change on agriculture, nutrition and health: A framework with a focus on fruits and vegetables** [version 2]. Wellcome Open Res 2017, 2:21 (doi: 10.12688/wellcomeopenres.11190.2). Reprinted with permission under the Creative Commons Attribution License.


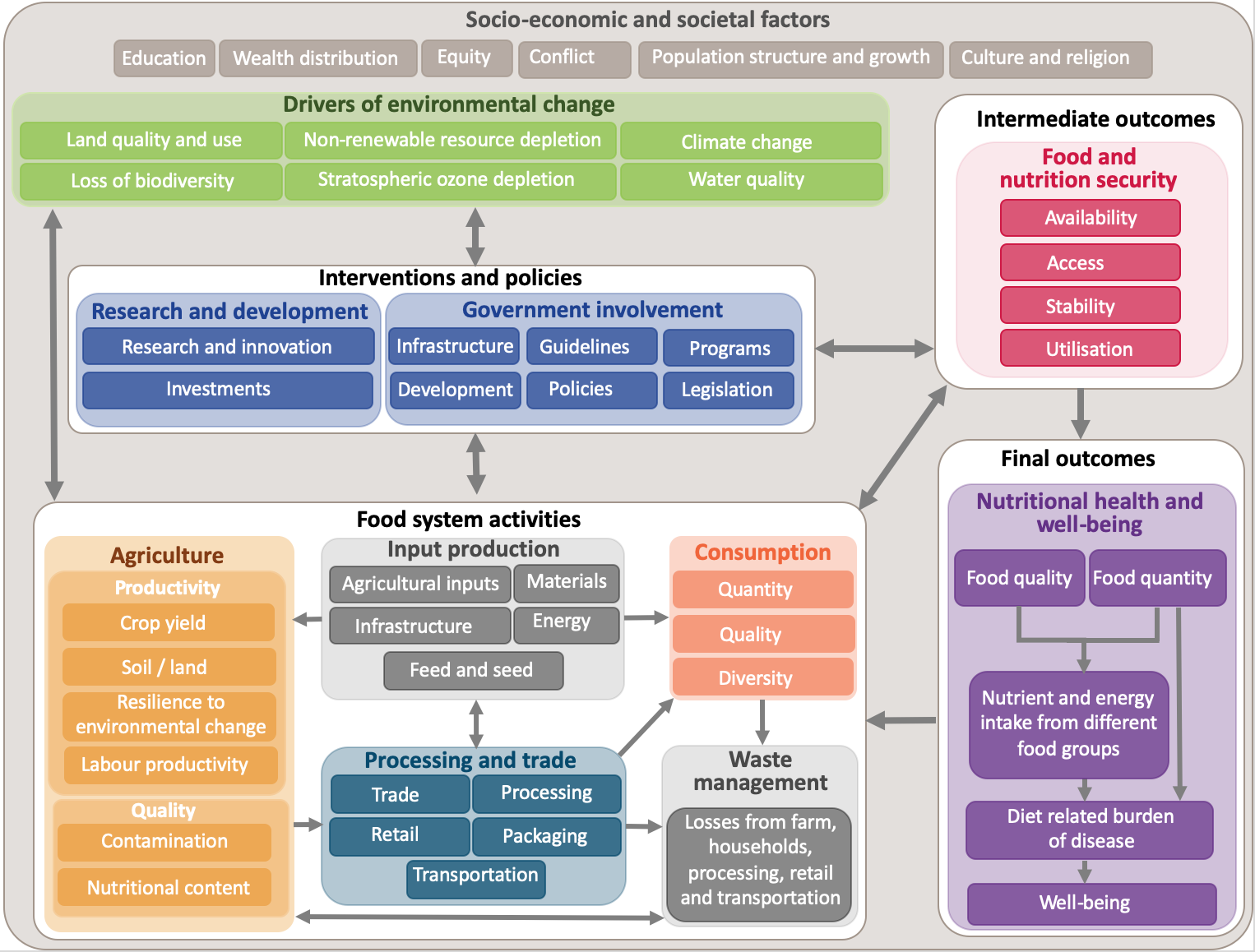


**Figure S1-Framework 5:** Masters, W. A. (2016). **The Economic Causes of Malnutrition**. In K. K. Eggersdorfer M, Cordaro JB, Fanzo J, Gibney M, Kennedy E, Labrique A, Steffen J, (Ed.), Good Nutrition: Perspectives for the 21st Century (pp. 92–104). Basel, Switzerland: Karger. Reprinted with permission.


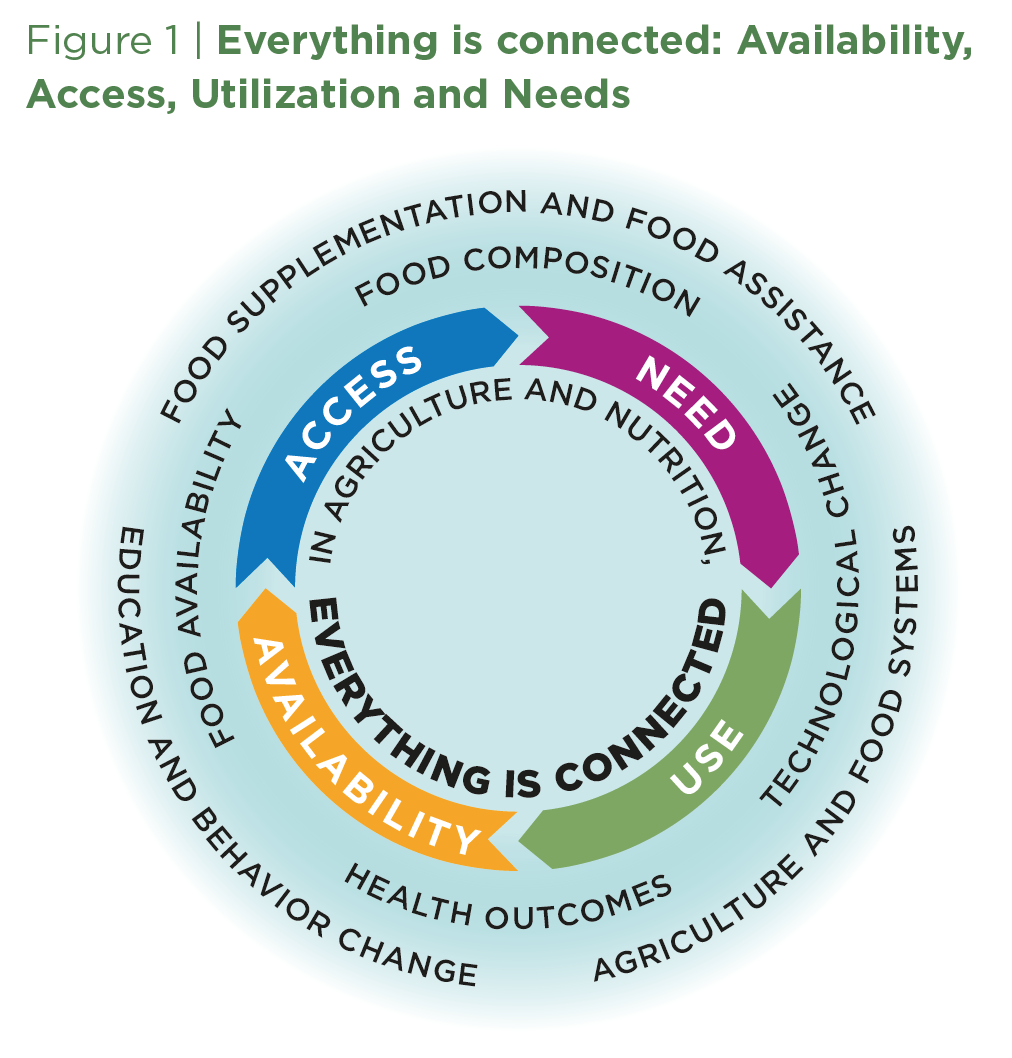


**Figure S1-Framework 6:** Global Panel. (2015). **Improved metrics and data are needed for effective food system policies in the post-2015 era: Technical Brief**. London, UK. Reprinted with permission under the Creative Commons Attribution License.


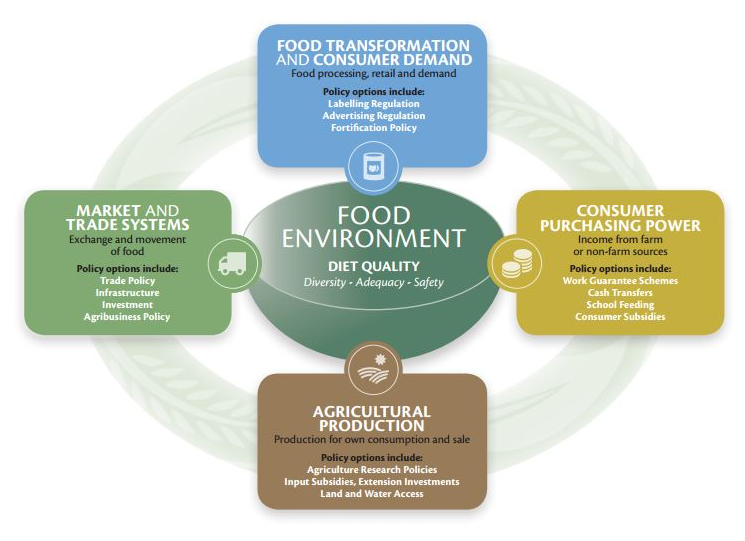


**Supplemental methods 1: Search strategy for published literature databases**

*CAB Abstracts listed below, with similar adapted for Web of Science*

Database: CAB Abstracts <1990 to 2018 Week 48>

Search Strategy: December 13 2018

--------------------------------------------------------------------------------

1 analytical methods/ or analysis/ or statistical analysis/ or methodology/ or experimental design/ or monitoring/ or measurement/ or data collection/ or models/ or mathematical models/ or environmental assessment/ or evaluation/ or performance indexes/ or program evaluation/ or social impact/ or environmental impact/ or impact/ or health impact assessment/

2 econometric models/ or econometrics/ or economic analysis/ or economic evaluation/ or economic theory/ or economic impact/ or cost effectiveness analysis/ or economic impact/

3 ("metrology" or "methods").id.

4 (new or original or unconventional or experimental or inventive or modern or advance* or innovat* or novel or introduc* or inaugurat* or launch* or recent* or up-to-date or updated or "not previously available" or emerging or validat* or adopt*).ti,ab.

5 ((new or original or unconventional or experimental or inventive or modern or advance* or innovat* or novel or introduc* or inaugurat* or launch* or recent* or up-to-date or updated or "not previously available" or emerging or validat* or adopt*) adj1 (method* or metric* or econometr* or metrolog* or measurement* or indicator* or meter* or module* or analy* or technolog* or technique* or application or device or tool or tools or toolkit*)).ti,ab.

6 ((1 or 2 or 3) and 4) or 5

7 (method* or metric* or econometr* or metrolog* or measurement* or indicator* or meter* or module* or analy* or technolog* or technique* or application or device or tool or tools or toolkit*).ti,ab.

8 1 or 2 or 3 or 7

9 exp agriculture/ or agricultural research/ or agronomy/ or farming/ or farming systems/ or exp horticulture/ or horticultural crops/ or market gardens/ or pastures/ or crop production/ or crop husbandry/ or crop losses/ or livestock/ or native livestock/ or animal husbandry/ or livestock farming/

10 (agriculture or agro-forestry or agroforestry or farming or horticulture or livestock or aquaculture or "fish farming" or ((food* or crop*) adj2 (produc* or grow* or cultivat* or rais* or harvest* or loss* or stor*)) or husbandry).ti,ab.

11 foods/ or food production/ or food safety/ or food processing/ or food storage/ or food storage losses/ or food environment/ or food deserts/ or food consumption/ or food policy/ or food security/ or food legislation/ or food marketing/ or food prices/

12 (food* adj2 (produc* or safety or process* or loss* or stor* or policy or policies or security or insecurity or consum* or environment or legislat* or market* or price or prices)).ti,ab.

13 exp nutrition/ or diets/ or nutrition research/ or nutrition surveys/ or nutritional assessment/ or nutritional state/ or nutrition programmes/ or nutrition security/ or community nutrition/ or nutrition policy/ or preventive nutrition/

14 (nutrition* or diet* or malnutrition or malnourish* or undernourish*).ti,ab.

15 or/9-14

16 6 and 15

17 limit 16 to yr="2008 -Current"

Annotation: New+Metrics+Ag/Nut/Food

18 8 and 15

19 limit 18 to yr="2008 -Current"

20 ("farm diversity score" or "functional diversity index" or ("household* food*" adj3 months) or ("women’s empowerment" adj2 agriculture) or ("food loss*" adj2 "supply chain*") or "global food loss* index" or "foodborne disease* burden" or "food safety score" or (coliform* adj2 milk) or (chloramphenicol adj2 residue*) or (diarrh* adj3 (child* or infant*) adj2 (prevalen* or epidemiolog* or distribut*)) or (water adj2 (distance* or collect*)) or (access* adj2 water adj2 (clean or improved)) or (cost* adj3 (diet* or "nutrient adequacy")) or (sale* adj2 (agricultur* or farm*) adj product*) or "household economy analysis" or "coping strateg* index" or ("household food insecurity" adj2 "access scale") or "food insecurity experience scale" or "household hunger scale" or "food consumption score" or "nutrition environment measurement tool* for stores" or (access* adj2 "healthy food") or "modified retail food environment" or "vulnerability and capacity assessment" or "nutrition* indicators for biodiversity" or "water footprint*" or ("soil quality" adj2 indicator*) or ("local authorit*" adj2 (response* or responsive*)) or (inclusive* adj2 participat* adj2 budget*) or (multi-stakeholder* adj2 partner*) or (conflict* adj2 interest adj2 safeguard*) or (access* adj2 "basic service*") or ("minimum dietary diversity" adj2 (women or child*)) or ("dietary diversity score" adj2 (women or household*)) or "minimum acceptable diet*" or "non-staple food energy" or "Shannon diversity" or "modified functional attribute diversity" or "nutrient* density score" or (nutrient* adj2 (intake or diversity or adequacy or availability)) or "nutrition* diversity" or ((diversity or bioversity) adj2 gradient*) or ("population share" adj2 "adequate nutrient*")).ti,ab,sh.

**Supplemental methods 2: Search strategy of grey literature databases, websites and project repositories**

| **Database** | **Search strategy and terms** | **Documents identified/screened before import** | **Documents imported for full screening** |
| --- | --- | --- | --- |
| AGRA | Hand-searched all documents | 0 | 0 |
| FAO Agris (via EBSCO) | See above, same as CAB Abstracts and Web of Science | 6324 | 5074 |
| ANH Academy, IMMANA | Hand-searched all documents | 15 grant pages | 24 |
| ATAI | Hand-searched all documents | 68 | 0 |
| BMGF | Hand-searched all documents | 0 | 0 |
| Campbell Collaboration | Agriculture | 0 | 0 |
| CENTRAL | ‘Agriculture’ | 1260 | 0 |
| CGIAR: A4NH | Hand-searched all documents | 4680 | 17 |
| CGIAR: IFPRI | ‘Agriculture’ OR ‘nutrition’ AND ‘measurement’ OR ‘method’ OR ‘matrix’, limits: 2009 year | 1175 | 16 |
| DFID Research for Development Outputs | ‘Agriculture’- 3399 hits  ‘Nutrition’- 2688 hits  (first 10 pages) | 6087 | 7 |
| 3ie | ‘Agriculture and rural development’ OR ‘child nutrition’ OR ‘nutrition’ | 302 studies screened, 0 eligible, search stopped | 0 |
| World Bank: IEG | ‘Agriculture’ - 64 hits and ‘health nutrition and population’ - 198 hits | 262 | 0 |
| World Bank: Documents and Reports | Documents selected by using:   - Sectors: agriculture, environment, rural development and the sub-sector nutrition - Document types: Publications and Research (environment working paper, IEG evaluation, issues in agriculture, policy research working paper, poverty and social policy working paper, and WBI working paper), and under Project Documents (impact evaluation report) - Studies were searched from 2008-18. | 3858 screened before import on title/abstract | 230 |
| USAID: Development Clearing House | ‘Agriculture’, limited by ‘Agriculture and Food Security’ as sector, and ‘Technical’ document type; used ‘sort by date’ function (first 30 pages) | 23,454 | 5 |
| USAID: What we do on agriculture and food security | Hand-searched all documents | 0 | 0 |
| USAID: Feed the Future Innovation Labs | Hand-searched all documents | 0 | 0 |
| USAID: AgriLinks | Hand-searched all documents using ‘agricultural productivity’ and ‘nutrition’ filters: <https://www.agrilinks.org> | 319 | 0 |
| USAID: FANTA | Hand-searched all documents [www.fantaproject.org](http://www.fantaproject.org)  <https://www.fantaproject.org/research> | 42 | 12 |
| USAID: Spring Nutrition | Hand-searched all documents using ‘Journal articles’ as publication type  [www.spring-nutrition.org](http://www.spring-nutrition.org) | 21 | 1 |
| Abbreviations: 3ie: International Initiative for Impact Evaluation; A4NH: Agricultural Development to Improve  Human nutrition and health; AGRA: Alliance for a Green Revolution in Africa; AGRIS: database; ANH: Agriculture, Nutrition and Health; ATAI: Agricultural Technology Adoption Initiative; BMGF: Bill and Melinda Gates Foundation; CENTRAL: Cochrane Central Register of Controlled Trials; DFID: United Kingdom Department for International Development; FANTA: Food and Nutrition Technical Assistance; FAO: Food and Agriculture Organization of the United Nations; IEG: Independent Evaluation Group; IFPRI: International Food Policy Research Institute IMMANA: Innovative Methods and Metrics for Agriculture and Nutrition Actions; USAID: United States Agency for International Development. | | | |

**The key publications we used for backward-citation tracking were:**

1. Girard AW, Self JL, McAuliffe C, Olude O. The effects of household food production strategies on the health and nutrition outcomes of women and young children: a systematic review. Paediatric and perinatal epidemiology. 2012;26 Suppl 1:205-22.

2. Global Panel. Improved metrics and data are needed for effective food system policies in the post-2015 era: Technical Brief. London, UK: Global Panel on Agriculture and Food Systems for Nutrition; 2015.

3. Hawkes C, Turner R, Waage J. Current and planned research on agriculture for improved nutrition: A mapping and a gap analysis. A report for DFiD: Leverhulme Centre for Integrative Research on Agriculture and Health (LCIRAH), Centre for Sustainable International Development, University of Aberdeen; 2012.

4. Herforth A, Nicolò GF, Veillerette B, Dufour C. Compendium of indicators for nutrition-sensitive agriculture. Rome, Italy: Food and Agriculture Organization of the United Nations; 2016.

5. Kadiyala S, Harris J, Headey D, Yosef S, Gillespie S. Agriculture and nutrition in India: mapping evidence to pathways. Ann N Y Acad Sci. 2014;1331:43-56.

6. Masset E, Haddad L, Cornelius A, Isaza-Castro J. Effectiveness of agricultural interventions that aim to improve nutritional status of children: systematic review. Bmj. 2012;344:d8222.

7. Ruel MT, Quisumbing AR, Balagamwala M. Nutrition-sensitive agriculture: What have we learned so far? Global Food Security. 2018;17:128-53.

8. Ruel MT, Alderman H, Maternal Child Nutrition Study Group. Nutrition-sensitive interventions and programmes: how can they help to accelerate progress in improving maternal and child nutrition? Lancet. 2013;382(9891):536-51.

9. Turner R, Hawkes C, Jeff W, Ferguson E, Haseen F, Homans H, Hussein J, Johnston D, Marais D, McNeill G, et al. Agriculture for improved nutrition: the current research landscape. Food Nutr Bull. 2013;34(4):369-77.

10. Webb P. Impact pathways from agricultural research to improved nutrition and health: literature analysis and research priorities. Rome: Food and Agriculture Organization and Geneva: World Health Organization. 2013.

**Supplemental methods 3: Detailed exclusion criteria for reports in the EGM**

- Tools, metrics or methods not applied to the domains that link agriculture, food systems and nutrition, as explicitly defined by the FAO or the conceptual frameworks included in Supplemental figure 1.
- Tools, metrics or methods developed or applied to agriculture-nutrition prior to 2008.
- Reports with no full-text reporting in English.
- In-vivo laboratory studies. If the subjects were animals for agricultural production, livelihoods or consumption, they were considered. If the animals were subjects as a proxy for humans or models for general interests (i.e. if the animals weren’t to sell or eat), they were excluded.
- In-vivo plant studies not explicitly related to agricultural production, land use, or other related themes. Like the exclusion for animal studies, if the plants were specifically mentioned in the context of agriculture or consumption, even if laboratory studies, they were considered. If the plants were a model of general cell function or not mentioned in relationship to the agriculture-ecology nexus, they were excluded.
- Discrete animal feeding experiments or analyses of soil, water, plants or animals that were not readily generalizable to the agriculture-food systems-nutrition pathway.
- Enhancement and therapeutic nutrition, such as parenteral nutrition in hospitalized patients, diets for niche chronic conditions, general dietary supplements (not including children and pregnant and lactating women), diet tools for weight loss. Studies on the prevention of overweight and obesity, or generally on healthy diets were included.
- In terms of the paper’s relationship to nutrition and nutritional proxies, food supplementation for communicable diseases (e.g. Tuberculosis, HIV), special groups such as hospital patients or athletes were all excluded.
- If innovations such as websites, applications, programs or technology were created but not described in any published report (either in our databases or in the grey literature repositories searched), then they were not captured in the map.

**Supplemental methods 4: Stage of development of tools, metrics and methods (full description)**

To construct a hierarchical coding for stage of development of tools, metrics and methods, we drew on literature on epidemiological indicator development as well as stages of innovation to create these four categories (31, 32):

1. Concept development and pilot: there is defined problem for which preliminary measures and methods were developed to understand the phenomenon or a pilot innovation has been described. The innovation is grounded in an understanding of the phenomenon.
2. Feasibility or internal validity: the innovation is feasible within a controlled setting and demonstrably can address the problem it intends to address in initial testing.
3. Demonstration and testing, external validity: the innovation captures what it intends to capture on a larger scale, across multiple settings or in less controlled environments.
4. Adoption, generalizability and wide-spread application: the innovation can be applied across multiple settings and contexts, captures what it intends to capture and is adopted by multiple stakeholders.

**Supplemental table 1: Coding structure of EGM and count of reports under each code**

| **Code field** | **COUNT** |
| --- | --- |
| Reports coded with a primary tool, metric or method | 904 |
| Secondary tools, metrics and methods | 133 |
|  |  |
| **Tool, metric or method category** |  |
| TOOLS |  |
| TOOLS: *Technology* | 164 |
| Tech tools: Instruments, devices, visual aids | 17 |
| Tech tools: Geospatial applications | 23 |
| Tech tools: Mobile, tablet, web apps, software | 66 |
| Tech tools: Biochemical tests (PCR, assays, rapid diagnostics) | 21 |
| Tech tools: Gene sequencing | 37 |
|  |  |
| TOOLS: *Survey, interview, research* | 56 |
| Other research tools: Quantitative | 47 |
| Other research tools: Qualitative | 9 |
| METRICS | 354 |
| METHODS | 330 |
| *Analysis, model* | 323 |
| *Research design* | 7 |
|  |  |
| **Domain** |  |
| Primary food production | 320 |
| Value chains, food transformation | 51 |
| Food safety | 45 |
| Economy | 54 |
| Markets | 30 |
| Food environments | 17 |
| Water, sanitation, hygiene | 206 |
| Ecology, sustainability, environment | 174 |
| Governance, food and trade policy | 84 |
| Power and conflict of interest | 9 |
| Food insecurity | 47 |
| Diet, nutrition, health | 383 |
|  |  |
| **Stage of development** |  |
| Stage 1: Concept development and pilot | 107 |
| Stage 2: Feasibility, efficacy or internal validity | 174 |
| Stage 3: Demonstration/testing, effectiveness, external validity | 134 |
| Stage 4: Adoption, generalizability and wide-spread application | 489 |
|  |  |
| **Setting or geographic application** |  |
| Global | 109 |
| Africa | 163 |
| Asia | 212 |
| Europe | 175 |
| Middle East, North Africa | 38 |
| North, Central America, Caribbean | 126 |
| Pacific/Oceana | 41 |
| South America | 41 |
|  |  |
| **Measurement unit** |  |
| Individual | 373 |
| Crop, product, animal, element | 138 |
| Household | 58 |
| Farm, field, plot, paddy | 75 |
| Community: city, sub-district, project, factory, school | 69 |
| District, sub-national | 89 |
| National | 77 |
| Regional | 12 |
| Global | 13 |
|  |  |
| **SUB-DOMAINS** |  |
| **Food production (FP)** | **314** |
| FP: Agriculture Assessment | 21 |
| FP:AA: Agriculture sector risk assessment | 15 |
| FP:AA: Women's Empowerment in Agriculture Index (WEAI) | 36 |
| FP: Agricultural capital and investment | 5 |
| FP: Agricultural pollution | 20 |
| FP: Agricultural systems, land use (Ag sys) | 34 |
| FP:Ag sys: Land governance assessment framework (LGAF) | 9 |
| FP:Ag sys: Nutritional Functional Diversity | 6 |
| FP:Ag sys: Production diversity | 10 |
| FP: Agroforestry | 6 |
| FP: Aquaculture | 17 |
| FP: Biofortification | 6 |
| FP:BF/MN: Biofortification Priority Index | 4 |
| FP: Climate Smart Agriculture (CSA) | 5 |
| FP: Connectivity, roads | 2 |
| FP: Farm/agriculture program management | 21 |
| FP: Livestock, Animal Source Foods (ASF) | 32 |
| FP: Soil | 40 |
| FP: Vulnerability, drought | 34 |
| FP: Yields, plant growth | 95 |
| FP: Other FP unique TMMs | 4 |
|  |  |
| **Water, sanitation, hygiene (WASH)** | 218 |
| WASH: Household water insecurity (HH WI) | 5 |
| WASH:HH WI: Household Water Insecurity Scale (HWIS) | 2 |
| WASH:HH WI: Other household water insecurity | 3 |
| WASH: Hydrology (Hydro) | 22 |
| WASH:Hydro: Aquacrop | 13 |
| WASH:Hydro: CropWat (FAO, New) | 11 |
| WASH:Hydro: Soil and Water Assessment (New SWAT) | 9 |
| WASH: Irrigation | 26 |
| WASH: Pricing for water | 2 |
| WASH: Water consumption | 4 |
| WASH: Water footprints (WF) | 146 |
| WASH:WF: Grey water footprints (GWF), water pollution | 18 |
| WASH:WF: LCA water footprints | 25 |
| WASH:WF: Virtual water trade, virtual footprints | 26 |
| WASH:WF: Water footprint assessment (WFN, post-2010) | 78 |
| WASH:WF: Other WASH unique TMMs | 20 |
| WASH: Water management | 17 |
| WASH: Water quality | 14 |
| WASH: Water scarcity/stress (WS) | 30 |
| WASH:WS: Available WAter Remaning (AWaRe) | 2 |
| WASH:WS: Blue water scarcity (Hoekstra et al. 2012) | 5 |
| WASH:WS: Green water scarcity (UNEP 2012) | 5 |
| WASH:WS: Water Scarcity Index (WSI) | 3 |
| WASH:WS: Water stress index (WSI)/Falkenmark indicator | 10 |
| WASH:WS: Other WS unique TMMs | 6 |
|  |  |
| **Ecology, sustainability, environment (Eco)** | 108 |
| Eco: Biodiversity | 10 |
| Eco: Climate change | 69 |
| Eco: Ecological Footprint Analysis (EFA) | 3 |
| Eco: Footprint family | 14 |
| Eco: Invasive species | 2 |
| Eco: Life cycle impact assessment (LCIA) | 13 |
| Eco: Sustainable diets | 9 |
| Eco: Other sustainability/planetary health | 7 |
|  |  |
| **Diet, nutrition, health (DNH)** | 409 |
| DNH: Adherence to dietary recommendations | 17 |
| DNH: Anthropometry | 3 |
| DNH: Anti-Microbial Resistance (AMR) | 2 |
| DNH: Children, breastfeeding, adolescents (U18) | 142 |
| DNH:U18: Australian Recommended Food Scores for Pre-schoolers (ARFS-P) | 2 |
| DNH:U18: Minimum Acceptable Diet for IYC (MAD) | 71 |
| DNH:U18: Minimum Dietary Diversity for IYC (MDD-C) | 72 |
| DNH:U18: Minimum Meal Frequency for IYC (MMF) | 55 |
| DNH:U18: ProPAN tool for complementary feeding programming | 3 |
| DNH:U18: WHO Infants and Young Children Feeding (IYCF) indicators | 93 |
| DNH:U18: Other U18 unique TMMs | 21 |
| DNH: Classification systems | 5 |
| DNH: Cooking, diet environment | 2 |
| DNH: Cost of diets (CoD) | 18 |
| DNH:CoD: Cost of the Diet (CotD) tool | 3 |
| DNH:CoD: Other CoD unique TMMs | 7 |
| DNH: Dietary software/web/apps (Tech) | 58 |
| DNH:Tech: Optifood | 11 |
| DNH:Tech: Self-administered web-based 24-h recall (R24W) | 2 |
| DNH:Tech: Web-based Dietary Assessment for School Children (WebDASC) | 2 |
| DNH:Tech: Other diet technology unique TMMs | 42 |
| DNH: Food insecurity, hunger, DD (FI, DD) | 84 |
| DNH:FI, DD: Adult (male) equivalents (AME) | 2 |
| DNH:FI, DD: FANTA Food Group Indicators (FGI) | 5 |
| DNH:FI, DD: Food Insecurity Experiences Scale (FIES) | 8 |
| DNH:FI, DD: Household Hunger Score (HHS) | 15 |
| DNH:FI, DD: Latin American Countries Food Security Scale (ELCSA) | 2 |
| DNH:FI, DD: Mean probability of adequacy (MPA) | 16 |
| DNH:FI, DD: Minimum Dietary Diversity for Women (MDD-W) | 19 |
| DNH:FI, DD: Months of Adequate Household Food Provisioning (MAHFP) | 8 |
| DNH:FI, DD: Other FI, DD unique TMMs | 14 |
| DNH: Healthy Diets (HD) | 95 |
| DNH:HD: Australian Recommended Food Score (ARFS) | 9 |
| DNH:HD: Dietary Quality Indices (DQI) | 13 |
| DNH:HD: Healthy Eating Indices (HEI) | 38 |
| DNH:HD: Healthy Lifestyle Diet Index (HLD-I) | 2 |
| DNH:HD: Mediterranean Diet Score (aMED/MED) | 5 |
| DNH:HD: Nutrient rich food index (NRI) | 5 |
| DNH:HD: NuVal Nutritional Scoring System | 2 |
| DNH:HD: Other HD unique TMMs | 15 |
| DNH: Illness, obesity prevention (NCDs) | 22 |
| DNH:NCDs: Dietary Approach to Stop Hypertension (DASH) | 5 |
| DNH:NCDs: Dietary Inflammatory Index (DII) | 11 |
| DNH:NCDs: Diet-Lifestyle Quality Index | 2 |
| DNH:NCDs: Polyphenol Antioxidant Content (PAC) Score | 2 |
| DNH:NCDs: Other NCD unique TMMs | 2 |
| DNH: Intake estimation | 34 |
| DNH: Micronutrients (MN) | 41 |
| DNH:MN: Fill the Nutrient Gap (FNG) | 4 |
| DNH:HD: Probability of Adequate Nutrient intake (PANDiet) | 15 |
| DNH:MN: Other MN unique TMMs | 9 |
| DNH: Models, algorithms | 14 |
| DNH: Sustainable diets/diets at pop level | 5 |
| DNH: Visual aids | 13 |
| SUB-TMM categories |  |
| Algorithms, simulation models | 27 |
| Artificial Intelligence (AI), Augmented Reality | 4 |
| Decision support | 67 |
| Geospatial tools, metrics, methods (secondary) | 38 |
| Network/DAG/complexity analysis (NA) | 62 |
| NA: Bayesian Networks | 56 |
| NA: Multiple Indicator Multiple Cause (MIMIC) models | 2 |
| NA: Neural networks (NN and Artificial NN) | 3 |
| NA: Systems dynamics | 3 |
| NA: Other network/DAG models | 4 |
| Participatory tools, metrics, methods | 21 |
|  |  |
|  |  |
| CROSS-CUTTING FILTERS |  |
| Carbon/energy (CE) | 30 |
| CE: Biofuels | 14 |
| CE: Carbon footprints | 11 |
| CE: Emergy assessment | 4 |
| CE: Other carbon/energy unique TMMs | 3 |
| Children | 165 |
| Disabilities and ill-health | 26 |
| Economics, inequality, poverty | 53 |
| Equity (PROGRESS+) | 81 |
| Equity: Place of residence | 11 |
| Equity: Race/ethnicity/culture/language | 0 |
| Equity: Occupation | 0 |
| Equity: Gender/sex | 47 |
| Equity: Religion | 0 |
| Equity: Education | 0 |
| Equity: SES | 27 |
| Equity: Social Capital | 5 |
| Equity: Plus | 5 |
| Food loss, waste | 7 |
| Microbiome (MB) | 34 |
| MB: Aquaculture | 5 |
| MB: Breastmilk | 1 |
| MB: Food | 1 |
| MB: Gut/fecal | 3 |
| MB: Soil | 23 |
| Private sector engagement | 2 |
| Shocks and humanitarian context | 26 |
| Technology | 160 |
|  |  |
|  |  |

**Supplemental table 2: List of unique tools, metrics and methods identified in reports**

*An Excel file with items that can be sorted by domain is available on request*

**METRICS**

**Table S2‑1 List of unique metrics by domain**

| **METRICS** | **Other Domain** | **Other Domain** |
| --- | --- | --- |
| **Diet, nutrition, health** |  |  |
| Adolescent Micronutrient Quality Index (AMQI) |  |  |
| Australian Diet Quality Index (Aussie-DQI) |  |  |
| Australian Recommended Food Score (ARFS) |  |  |
| Australian Recommended Food Scores for Pre-schoolers (ARFS-P) |  |  |
| Baltic Sea Diet Score |  |  |
| Brazilian Healthy Eating Index Revised (hypothesis-driven) for diet analysis |  |  |
| Breakfast Quality Index (BQI) for children and adolescents |  |  |
| Exclusive Breastfeeding Social Support Scale (EBFSS) |  |  |
| Food Standards Agency Nutrient Profiling System Dietary Index (FSA-NPS DI) in French middle-aged adults |  |  |
| Carbohydrate (CHO) Quality Index (CQI) |  |  |
| Child Feeding Index (CFI) |  |  |
| Children's Dietary Life Safety (CDLS) Index |  |  |
| Children's Index of Diet Quality (CIDQ) |  |  |
| Cost of Recommended Diet (CoRD) |  |  |
| Diet Quality Index based on Food-Based Dietary Guidelines (FBDG) of Denmark |  |  |
| Diet Quality Index for Adolescents |  |  |
| Diet quality score for preschool children |  |  |
| Diet-lifestyle quality index |  |  |
| Dietary Approach to Stop Hypertension (DASH) score |  |  |
| Dietary diversity score in children 5-8 year-olds |  |  |
| Dietary Inflammatory Index (DII) |  |  |
| Dietary Quality Indices (DQI) |  |  |
| Eating Choices Index (ECI) |  |  |
| Energy Density Score (EDS) |  |  |
| FANTA Food Group Indicators (FGI) |  |  |
| Food Frequency Index (FFI) |  |  |
| Food Rating Scale in food service |  |  |
| Food-based diet quality score |  |  |
| Forest Food Consumption Score (FFCS) |  |  |
| French Programme National Nutrition Sante-Guideline Score (mPNNS-GS) |  |  |
| Fruit And Vegetable Variety Score (FAVVA) |  |  |
| Healthy Dietary Habits Index (HDHI) |  |  |
| Healthy Dietary Habits Score for Adolescents (HDHS-A) |  |  |
| Healthy Eating Indices (HEI) |  |  |
| Healthy Food Availability Indices (HFAI) |  |  |
| Healthy Food Diversity (HFD) index |  |  |
| Healthy Food Intake Index (HFII) |  |  |
| Healthy Lifestyle Diet Index (HLD-I) |  |  |
| Healthy Nutrition Score for Kids and Youth (HuSKY) |  |  |
| Infant and Young Child Feeding Indicators (WHO revised 2008) |  |  |
| Malnutrition Gap measure |  |  |
| Mediterranean Diet Quality Index (KIDMED) |  |  |
| Mediterranean Diet Score (aMED/MED) |  |  |
| Mexican Diet Quality Index (ICDMx) |  |  |
| Minimum Acceptable Diet for Infants and Young Children (MAD) |  |  |
| Minimum Dietary Diversity for Infants and Young Children (MDD-C) |  |  |
| Minimum Meal Frequency for Infant and Young Child Feeding (MMF) |  |  |
| Non-Recommended Food Score (non-RFS) |  |  |
| Nutrient density score of carbohydrate-rich foods |  |  |
| Nutrient rich food index |  |  |
| Nutritious Food Price Index (NPI) |  |  |
| NuVal Nutritional Scoring System |  |  |
| Overall Nutritional Quality Index (ONQI) |  |  |
| Polyphenol Antioxidant Content Score (PAC-Score) |  |  |
| Pre-schoolers Diet-Lifestyle Index (PDL-Index) |  |  |
| Prime Diet Quality Score (PDQS) |  |  |
| Probability of Adequate Nutrient intake (PANDiet) |  |  |
| ProPAN tool for complementary feeding programming |  |  |
| Recommendation Compliance Index (RCI) |  |  |
| Recommended Food Score (RFS) |  |  |
| Food Group Diversity Indicators (FGIs) |  |  |
| Traditional food diversity score |  |  |
| Unhealthy Food Availability Indices (UFAI) |  |  |
| Women's Empowerment in Nutrition (WENI) |  |  |
| **Primary food production** |  |  |
| Nutritional Functional Diversity | DNH | Ec, Su, En |
| Fishery performance indicators | Ec, Su, En | E |
| Biofortification Priority Index (BPI) | Ec, Su, En | Gov, Pol |
| Climate Smart Agriculture Policy Index (CSA-Pol Index) | Ec, Su, En | Gov, Pol |
| Climate Smart Agriculture Results Index (CSA-Res Index) | Ec, Su, En | Gov, Pol |
| Climate Smart Agriculture Technology Index (CSA-Tech Index) | Ec, Su, En | Gov, Pol |
| Agricultural Systems Vulnerability Index (ASVI) | Ec, Su, En |  |
| Drought Vulnerability Index (DVI) | Ec, Su, En |  |
| Index of biological boil quality (IBQS, *Indice Biotique de la Qualité du Sol*) | Ec, Su, En |  |
| Maximum Water Capacity (MWC) | Ec, Su, En |  |
| Soil Dynamic Quality Index (S-DQI) derived through fuzzy logic | Ec, Su, En |  |
| Soil Fertility Quality Index (SFQI) | Ec, Su, En |  |
| Soil Quality Index (SQI) | Ec, Su, En |  |
| Soil quality index for typical temperate, maritime grassland management | Ec, Su, En |  |
| Support Area (SA) index (for environmental load) | Ec, Su, En |  |
| Strawberry irrigation metrics [Relative Irrigation Supply (RIS), Strawberry Irrigation Water Applied (SWA), Strawberry Water Footprint Applied (SWFA)] | WASH |  |
| Innovation indicators for developing-country agriculture |  |  |
| Land Management Index (LMI) decision support tool |  |  |
| Production diversity |  |  |
| Women's Empowerment in Agriculture Index (WEAI, ProWEAI, A-WEAI) |  |  |
| Women's Empowerment in Livestock Index (WELI) |  |  |
| **Water, sanitation, hygiene** |  |  |
| Pollution Water Indicator (PWI) | Ec, Su, En |  |
| Water Ecotoxicological Footprint | Ec, Su, En |  |
| Water Self Sufficiency (WSS) | Ec, Su, En |  |
| Blue water scarcity (Hoekstra et al. 2012) | Mixed |  |
| Agricultural Water Stress Index (AWSI) | FP |  |
| Crop Water Productivity (CWP) Index | FP |  |
| Water Scarcity Index (WSI) | (Ec, Su, En) |  |
| Drinking Water Nutritional Quality Index (DWNQI) |  |  |
| Freshwater Provision Index (FPI) |  |  |
| Green water scarcity (UNEP 2012) |  |  |
| Household Water Insecurity Access Scale (HWIAS) |  |  |
| Household Water InSecurity Experiences (HWISE) |  |  |
| Household Water Insecurity Scale (HWIS) |  |  |
| Locally developed water insecurity scale |  |  |
| Water Quality Index (WQI) |  |  |
| Falkenmark indicator |  |  |
| **Food insecurity** |  |  |
| Food security measurement tool |  |  |
| Dynamic food insecurity indicator |  |  |
| Food Access Survey Tool (FAST) |  |  |
| Food Insecurity Experiences Scale (FIES) |  |  |
| Household Hunger Score (HHS) |  |  |
| Household Food Security Survey Module |  |  |
| Latin American and Caribbean Food Security Scale (ELCSA) |  |  |
| Mean Probability of Adequacy (MPA) |  |  |
| Minimum Dietary Diversity for Women (MDD-W) |  |  |
| Months of Adequate Household Food Provisioning (MAHFP) |  |  |
| Listeria Food Exposure Score (LFES) |  |  |
| **Economy** |  |  |
| Starchy Staples Expenditure Ratio (SSEXR) | F Ins |  |
| Exact Price Index (EPI) | F Env |  |
| Cost of Dietary Diversity (CoDD) | M |  |
| Cost of Nutrient Adequacy (CoNA) indicator | M |  |
| Basic Needs Poverty Line (BNPL) |  |  |
| Human Opportunities Index (HOI) |  |  |
| **Ecology, sustainability, environment** |  |  |
| Bayesian Risk Index (BRI) | F safety |  |
| Environmental risk score using multiple indicators |  |  |
| Vigneto sustainability indicator |  |  |
| VIGOR index [Vigor (V), Net Primary Production (NPP), O (Organization: area proportion of nature ecosystem, Shannon Diversity Index (SHDI), Contagion Index (CONTAG), R (Resilience: ecology elasticity), and S (Service: water conservation, soil conservation)] |  |  |
| **Food environments** |  |  |
| Food waste performance indicator FRESH number | Ec, Su, En |  |
| **Markets** |  |  |
| Cost of staple and non-staple calories from Markets | F Env | DNH |
| **Abbreviations:** DNH: Diet, nutrition, health; E: Economy; Ec, Su, En: Ecology, sustainability, environment; F Env; Food environments; F Ins: Food insecurity; F Safe: Food safety; FP: Primary food production; Gov, Pol: Governance, food and trade policy; M: Markets; VC, FT: Value chains, food transformation; WASH: Water, sanitation, hygiene | | |
| Domains in brackets are coded on some reports but not all | | |

**TOOLS**

**Table S2‑2 List of unique instrument, devices and visual aid tools by domain**

| **INSTRUMENTS, DEVICES, VISUAL AIDS** | **Other Domain** |
| --- | --- |
| **Diets, nutrition, health** |  |
| Augmented Reality (AR) for dietary knowledge and intake |  |
| DietBytes image-based dietary assessment method |  |
| Food photography 24-h recall method (FP 24-hR) |  |
| Food photography manual |  |
| Food quantification picture book for children |  |
| Healthy lifestyle guide pyramid educational tool for children and adolescents |  |
| Photographic food records |  |
| Self-Completed Recall and Analysis of Nutrition (SCRAN24) for use with children |  |
| Visual estimation methods for children |  |
| **Primary food production** |  |
| Diffusive Gradients in Thin films (DGT) (new application to Cu, P and Zu) | Ec, Su, En |
| Magnetic leaf patch clamp pressure probe (ZIM-probe) |  |
| Motion graphics for determining soil quality | Ec, Su, En |
| Computer vision system |  |
| Remote sensing of water quality | Ec, Su, En |
| Unmanned Aerial Vehicle (UAV)-Assisted Measurement System (UAMS) for water quality |  |
| **Food safety** |  |
| Electrochemical 'DEP-On-Go' sensor to determine total viable counts (TVCs) of live microbes in food |  |
| **Abbreviations:** DNH: Diet, nutrition, health; E: Economy; Ec, Su, En: Ecology, sustainability, environment; F Env; Food environments; F Ins: Food insecurity; F Safe: Food safety; FP: Primary food production; Gov, Pol: Governance, food and trade policy; M: Markets; VC, FT: Value chains, food transformation; WASH: Water, sanitation, hygiene | |

**Table S2‑3 List of unique geospatial application tools by domain**

| **GEOSPATIAL APPLICATIONS** | **Other Domain** | **Other Domain** |
| --- | --- | --- |
| **Primary food production** |  |  |
| Applying multiple imputation for predicting missing GPS-based land area measures in household surveys |  |  |
| CityCrop model | WASH |  |
| Digital Elevation Models (DEMs) and global positioning system-based guidance systems for seed planting |  |  |
| Geographically explicit quantification of current and future use of blue and green water consumption in agriculture | F Env |  |
| Geospatial approach for agroforestry suitability mapping | Ec, Su, En |  |
| High resolution-data and a GIS system for Water Footprint (WF) of agricultural crops |  |  |
| Hydrogeological GIS-based mapping | WASH |  |
| Land Suitability Analysis (LSA) | Ec, Su, En |  |
| REGularized canopy reFLECtance (REGFLEC) image-based inverse canopy radiative transfer modelling system |  |  |
| Remote sensing hydrologic model |  |  |
| Self-learning cruising water quality monitoring system | WASH |  |
| Self-Organizing Map (SOM) neural image classification technique |  |  |
| Soil Quality Index (SQI) based on geospatial data |  |  |
| Spatial analysis of transport infrastructure and agriculture | M | VC, FT |
| **Water, sanitation, hygiene** |  |  |
| Integrated Watershed Management Model (IWMM): GIS and GPS for characterizing land use systems contributing to watershed pollution from agriculture | FP |  |
| Night-time satellite observed lit area for assessment of global water footprint |  |  |
| PHYTOPIXAL: GIS-based decision tool with multiple indicators to determine contamination risks |  |  |
| Rainwater Harvesting Decision Support System (RHADESS) |  |  |
| Weighted linear combination, Boolean techniques within GIS environment to select water harvesting ponds |  |  |
| **Food safety** |  |  |
| Geospatial risk model for food-borne pathogens |  |  |
| **Markets** |  |  |
| Rural Access Index (RAI) | E |  |
| Urban food diagnostic and metric framework | F Env |  |
| **Value chains, food transformation** |  |  |
| Australian Algal Cultivation-Spatial Location Model (AAC-SLM) for GIS |  |  |
| **Abbreviations:** DNH: Diet, nutrition, health; E: Economy; Ec, Su, En: Ecology, sustainability, environment; F Env; Food environments; F Ins: Food insecurity; F Safe: Food safety; FP: Primary food production; Gov, Pol: Governance, food and trade policy; M: Markets; VC, FT: Value chains, food transformation; WASH: Water, sanitation, hygiene | | |

**Table S2‑4 List of unique mobile, tablet, web apps and software tools by domain**

| **MOBILE, TABLET, WEB APPS, SOFTWARE** | **Other Domain** | **Other Domain** |
| --- | --- | --- |
| **Diets, nutrition, health** |  |  |
| Foodbook24 | Ec, Su, En | WASH |
| Optifood | (M) |  |
| Web-based nutritional knowledge questionnaire for children and adolescents | WASH |  |
| Automated Self-Administered 24-hour diet recall (ASA24) |  |  |
| Automated, self-administered web-based 24-h recall (R24W) |  |  |
| Calculator of Inadequate Micronutrient Intake (CIMI) |  |  |
| CATI - mobile phones for diet data collection |  |  |
| DIAL software |  |  |
| Diet Assess & Plan (DAP) platform |  |  |
| Diet-A mobile application |  |  |
| Digital Image-based Food Record (DIFR) method |  |  |
| Electronic Diet History of ENRICA (DH-E) |  |  |
| Electronic mobile-based food record (e-CA) |  |  |
| Evernote app-based electronic food diary |  |  |
| Healthy Eating Quiz (HEQ) |  |  |
| INTAKE24 web-based 24-hour dietary recall system |  |  |
| Lives Saved Tool (LiST) |  |  |
| Mobile device (for example, PDA, mobile phone) food record |  |  |
| Mobile vitamin D calculator application |  |  |
| Multiple Micronutrient Survey (MMS) cost-benefit tool |  |  |
| Multiple Source Method (MSM) program |  |  |
| My Meal Mate (MMM) |  |  |
| MyFitnessPal (MFP) dietary intake records |  |  |
| Myfood24 |  |  |
| New Interactive Nutrition Assistant - Diet in India Study of Health (NINA-DISH) |  |  |
| Novel Assessment of Nutrition and Ageing (NANA) |  |  |
| NutPlan Nutritional software |  |  |
| Nutricam Dietary Assessment Method (NuDAM) |  |  |
| Online Food Frequency Questionnaires (FFQ) |  |  |
| Oxford WebQ |  |  |
| PDA-based food diary with food photographs |  |  |
| Pictorial, web-based version of the NCI diet history questionnaire (Web-PDHQ) |  |  |
| ProPAN software |  |  |
| Remote Food Photography Method (RFPM) |  |  |
| Self-administered, computerised, 24-h DR (PAC24) for children |  |  |
| Smartphone-assisted 3-day, 24-hour recall for beverage intake |  |  |
| Smartphone-based photographic food recording approach |  |  |
| Smartphone-based tool to collect thrice-repeated 24 h dietary recall data |  |  |
| SMS questionnaires for drinking water consumption |  |  |
| Technology Assisted Dietary Assessment (TADA) system, mobile food record (mFR) 24-h dietary recall via FaceTime (24 HR-FT) |  |  |
| Web-based Computer-Assisted Personal Interview System (CAPIS) |  |  |
| Web-based Dietary Assessment for School Children (WebDASC) |  |  |
| Web-based Food Frequency Questionnaire |  |  |
| Web-based, self-administered Dietary Record (DR) tool |  |  |
| **Primary food production** |  |  |
| BioGrace carbon tool | Ec, Su, En |  |
| Cool Farm Tool (CFT) | Ec, Su, En |  |
| Agrifood decision tool |  |  |
| Water Footprint tool |  |  |
| Web-based tool using LOAD ESTimator (LOADEST) |  |  |
| **Water, sanitation, hygiene** |  |  |
| Aquacrop | Mixed |  |
| CropWat (New, FAO) | FP | Ec, Su, En |
| **Markets** |  |  |
| Cost of the Diet (CotD) tool | E | DNH |
| **Abbreviations:** DNH: Diet, nutrition, health; E: Economy; Ec, Su, En: Ecology, sustainability, environment; F Env; Food environments; F Ins: Food insecurity; F Safe: Food safety; FP: Primary food production; Gov, Pol: Governance, food and trade policy; M: Markets; VC, FT: Value chains, food transformation; WASH: Water, sanitation, hygiene | | |
| Domains in brackets are coded on some reports but not all | | |

**Table S2‑5 List of unique biochemical tools by domain**

| **BIOCHEMICAL TESTS** | **Other Domain** |
| --- | --- |
| **Food safety** |  |
| Liquid-chromatography-electrospray tandem mass spectrometry multiclass method for determination of 45 veterinary compounds belonging to 9 different antibiotic groups | Ec, Su, En |
| Ridascreen (R-Biopharm) ELISA kit for Chloramphenicol (CAP) | Ec, Su, En |
| Isolating bacteriophage from water based on electropositive silica gel particles (ESPs) method | WASH |
| Bioelectrochemical immunoassay method to detect Chloramphenicol (CAP) residues in milk |  |
| Chloramphenicol (CAP) specific aptamer and real-time fluorescent quantitative PCR (qRT-PCR) |  |
| Combination of a selective Solid-Phase Extraction (SPE) and dispersive liquid-liquid microextraction (chloramphenicol in water, milk, honey, and urine) |  |
| Dual-Label Time-Resolved Chemiluminescent Multiplexed Immuno Assay (DLTRC-MIA) for detecting antibiotics in milk |  |
| Gold Immuno-Chromatographic Assay (GICA) for detection of multiple (4) antibiotics in food (milk, honey) |  |
| HPLC with UV detection-based assay for chloramphenicol (CAP) residues (for aquatic products) |  |
| Immuno-Affinity Column (IAC) clean-up coupled with Liquid Chromatography-tandem Mass Spectrometry (LC-MS/MS) method |  |
| Liquid Chromatography-High Resolution Mass Spectrometry (LC-HRMS) for detecting chloramphenicol in meat |  |
| Mesofluidic immunoassay system for chloramphenicol detection in foods |  |
| MicroSnap Coliform and E. coli test system |  |
| Molecular imprinting technique combined with solid-phase extraction |  |
| Multi-residue method for detecting antibiotic residues in aquaculture |  |
| Polymerase Chain Reaction (PCR) assay Species-specific for Listeria |  |
| qPCR assay to quantify environmental Acanthamoeba and disinfection efficacy |  |
| Time-Resolved FluoroImmuno Assay (TRFIA) technique to detect chloramphenicol (CAP) contamination in food |  |
| **Primary food production** |  |
| LAMP assay to specifically identify V. parahaemolyticus causing AHPND (V. parahaemolyticus AHPND) |  |
| Neobenedenia-specific real-time quantitative polymerase chain reaction (qPCR) assay |  |
| **Ecology, sustainability, environment** |  |
| HPLC and fluorescence method for Avermectin detection | F Safe |
| **Abbreviations:** DNH: Diet, nutrition, health; E: Economy; Ec, Su, En: Ecology, sustainability, environment; F Env; Food environments; F Ins: Food insecurity; F Safe: Food safety; FP: Primary food production; Gov, Pol: Governance, food and trade policy; M: Markets; VC, FT: Value chains, food transformation; WASH: Water, sanitation, hygiene | |

**Table S2‑6 List of unique gene sequencing tools by domain**

| **GENE SEQUENCING** | **Other Domain** | **Other Domain** |
| --- | --- | --- |
| **Primary food production** |  |  |
| Metabarcoding, 16S rRNA, 18S rDNA, gp60, 454 pyrosequencing, Basic Local Alignment Search Tool (BLASTn), ITS-2 and ITS-1 fragments | Ec, Su, En | DNH |
| **Abbreviations:** DNH: Diet, nutrition, health; E: Economy; Ec, Su, En: Ecology, sustainability, environment; F Env; Food environments; F Ins: Food insecurity; F Safe: Food safety; FP: Primary food production; Gov, Pol: Governance, food and trade policy; M: Markets; VC, FT: Value chains, food transformation; WASH: Water, sanitation, hygiene | | |

**Table S2‑7 List of unique survey, interview and research tools by domain**

| **SURVEY, INTERVIEW, RESEARCH** | **Other Domain** |
| --- | --- |
| **Diets, nutrition, health** |  |
| 16-item Food Intake Questionnaire (16-FIQ) |  |
| Child And Diet Evaluation Tool (CADET) |  |
| Compendium of Early Childhood Development tools: - UNICEF Multiple Indicator Cluster Surveys (MICS), Early Childhood Development Module - Family Care Indicators (FCI) (Kariger et al. 2012) - Parenting Interactions with Children: Checklist of Observations Linked to Outcomes (PICCOLO) (Roggman et al. 2013) - Observation of Mother-Child Interactions (OMCI) (Rasheed and Yousafzai 2015) - Classroom Assessment Scoring System (CLASS) (Pianta, La Paro, and Hamre 2008) - Measure of Early Learning Environments (MELE) | F Ins |
| DASH questionnaire |  |
| Diet Quality Screener (sDQS) brief Mediterranean Diet Screener (bMDSC) |  |
| Diet questionnaire to assess habitual diet and phyto-oestrogen intake |  |
| Dietary Questionnaire for Epidemiological Studies (DQESV2) |  |
| Electronic-Nutrition Literacy Tool (e-NutLiT) |  |
| European Food Consumption Validation (EFCOVAL) trans-European food consumption method | E |
| EURRECA (EURopean micronutrient RECommendations Aligned) toolbox | VC, FT |
| Healthy Home Survey | Ec, Su, En |
| Iterative algorithm to identify the best fuzzy dietary pattern constrained by energy and nutrients |  |
| Longitudinal assessment of nutrient intake in infants and young children |  |
| Meal recall questionnaire for populations with poor literacy |  |
| Mediterranean Diet Adherence Screener (MEDAS) | F Ins |
| Method for assessing individual dietary intake from common-plate meals | E |
| Modification of linear programming for food-based recommendations |  |
| Multiple Micronutrient Supplementation (MMS) cost-benefit tool |  |
| MyPlate classification system | WASH |
| NOVA processed foods classification system |  |
| NutricheQ Questionnaire for toddlers |  |
| Nutrient-profiling algorithm based on HEI score |  |
| NutriQuid (NQ) self-administered, structured food record |  |
| Online 121-item Food Recall Checklist (FoRC) |  |
| Pre-coded food diary |  |
| Short dietary questionnaire (23 items) |  |
| **Ecology, sustainability, environment** |  |
| Multi-Attribute Value Theory (MAVT)-based decision support tool (DST) for facilitating sludge treatment decisions |  |
| Composite database of sustainable diet metrics for common foods |  |
| DESTISOL decision support system | E |
| Nutrition environment measurement tool for stores (NEMS-S) |  |
| Nutrition Environment Measures Survey in Convenience Stores (NEMS-CS) adapted Food Store Observation Form from the Bridging the Gap Community Obesity Measures Project (BTG-COMP) | DNH |
| Rapid Emergency Food Security Assessment (EFSA) survey |  |
| **Primary food production** |  |
| Deriving post-harvest loss estimates from Living Standards Measurement Study-Integrated Surveys on Agriculture initiative (LSMS-ISA) |  |
| Early warning tools for drought response in social safety nets: - Livelihoods, Early Assessment and Protection (LEAP) tool (2008) - Livelihood Impact Analysis Sheet (LIAS) (2008) - hotspots assessments conducted by the Early Warning and Response Directorate (EWRD) |  |
| Machine learning algorithms to predict insemination outcomes of cows |  |
| Marginal cost curves (MCCs) for water footprint assessment |  |
| Non-linear programming for feed formulation |  |
| Production diaries |  |
| Rural Household Multi-Indicator Survey (RHoMIS) | E |
| **Economy** |  |
| Food purchase patterns tool to measure food security |  |
| Linear programming to determine lowest cost diet using locally consumed foods and meeting micronutrient needs | DNH |
| **Food environments** |  |
| Food Access Survey Tool (FAST) |  |
| Food Preparation Checklist (FPC) |  |
| **Governance, food and trade policy** |  |
| SENS algorithm, a new nutrient profiling system |  |
| **Value chains, food transformation** |  |
| Fortification Assessment Coverage Toolkit (FACT) |  |
| **Water, sanitation, hygiene** |  |
| Ecoinvent |  |
| **Abbreviations:** DNH: Diet, nutrition, health; E: Economy; Ec, Su, En: Ecology, sustainability, environment; F Env; Food environments; F Ins: Food insecurity; F Safe: Food safety; FP: Primary food production; Gov, Pol: Governance, food and trade policy; M: Markets; VC, FT: Value chains, food transformation; WASH: Water, sanitation, hygiene | |

**Table S2‑8 List of unique qualitative tools by domain**

| **QUALITATIVE TOOLS** | **Other Domain** | **Other Domain** |
| --- | --- | --- |
| **Primary food production** |  |  |
| Climate analogues - tool to help people visualize future climate and environment | Ec, Su, En | Gov, Pol |
| Combination of Agent-Based Modelling (ABM), Bayesian Belief Networks (BBNs), Opinion Dynamics Models (ODM) | Ec, Su, En |  |
| Decision tool for nitrogen fertilizer application |  |  |
| Qualitative application of Women's Empowerment in Agriculture Index (WEAI) domains | M |  |
| Water Delivery for Irrigation (WaDI) model | WASH |  |
| **Diets, nutrition, health** |  |  |
| Brazilian dietary diversity scoring system for plates |  |  |
| Decision tool for Multi-Stakeholder Partnerships (MSPs) | Gov, Pol |  |
| **Ecology, sustainability, environment** |  |  |
| Community concept drawing |  |  |
| **Abbreviations:** DNH: Diet, nutrition, health; E: Economy; Ec, Su, En: Ecology, sustainability, environment; F Env; Food environments; F Ins: Food insecurity; F Safe: Food safety; FP: Primary food production; Gov, Pol: Governance, food and trade policy; M: Markets; VC, FT: Value chains, food transformation; WASH: Water, sanitation, hygiene | | |

**METHODS**

**Table S2‑9 List of unique analysis and model methods by domain**

| **ANALYSIS, MODEL** |
| --- |
| Adding CO2 emissions from feeding and transportation to classic economic model |
| Adult Male Equivalents (AME) |
| AGEVAR MODE statistical model - improved method to estimate usual intake by age group |
| Agricultural sector risk assessment |
| Agricultural technology adoption model |
| Algorithm for Fast Food Restaurant classification |
| Anthropometry as a proxy for individual poverty |
| Applying latent class analysis to ecological footprint labelling of consumer products |
| Applying social context to formal rational-actor theories |
| Available WAter Remaning (AWaRe) |
| Bayesian Networks |
| BioSpatial H2O (system dynamics modelling and database framework) for biomass feedstock |
| Biotracing model Quantitative Microbial Risk Assessment (QMRA) |
| Carbon and water footprints |
| Combination model: neural network algorithm, classification regression tree algorithm, Bayesian network algorithm |
| Combining indicators of underlying determinants of malnutrition and comparing to anthropometry in a new way |
| Combining Multiple Source Method (data-driven) |
| Consumptive Water reduction from changing diets and food loss |
| Crop virtual water assessment |
| Cross-Sectoral Footprints (water, energy, food, land) |
| Decision analysis tools for policy planning |
| Decision analysis tools to model national agricultural development plans |
| Dietary gap assessment (plausible healthy diet for nation + FAO food balance sheets) |
| DIETCOST programme - modelling tool |
| Disaggregated and highly-detailed crop model based on climate change models |
| Discrete choice experiment Random utility models, Random choice models |
| DSSAT (CERES-Rice) crop growth model |
| Dynamic and stochastic simulation model for water footprint of sheep grazing |
| Dynamic Computable General Equilibrium (DCGE) model for Tanzania |
| Dynamic viability model to plan soil quality restoration |
| Ecological Footprint Analysis (EFA) |
| Economic risk analysis of agricultural practices, farm simulation model |
| Economy-wide computable general equilibrium model |
| Emergy assessment |
| Estimating precipitation using soft computing methods and GARCH time series model |
| Field-scale resource Interactions, use Efficiencies and Long-term soil fertility Development (FIELD) model |
| Fill the Nutrient Gap (FNG) |
| Food-Energy (FEW) nexus analysis framework |
| Foreign Direct Investment (FDI) to analyse nutrient intakes across family roles measuring effect of economic openness on intra-household food allocation |
| Forest, Agriculture, and Biofuels in a Land use model with Environmental services (FABLE) |
| Freshwater Fish Injurious/Invasive Species Risk Assessment Model (FISRAM) |
| Gaussian Graphical Models (GGMs) |
| Generic Impact Scoring System (GISS) |
| Grey water footprint analysis |
| Historical energy cost accounting (including digestion, pregnancy, and lactation) |
| hybrid approach for assessing bioenergy potentials for regions with diverging economic, social and ecological systems |
| Hydro-economic Multi-Regional Input-Output (MRIO) Analysis |
| HYDRUS (2D/3D) model |
| Inequality Footprint |
| INFORMAS indicators and methods |
| Input-output structural decomposition analysis |
| Integrated Farm System Model for environmental footprints |
| Integrated hydro-economic dynamic optimization model |
| Land Degradation Surveillance Framework (LDSF) |
| Land footprints for intensification of livestock production |
| Land Governance Assessment Framework (LGAF) |
| Landscape DNDC biogeochemical model |
| Leaf Area Index (LAI) time-series mapping |
| Life Cycle (Impact) Analysis (LCA) for water footprint |
| Life Cycle Impact Assessment (LCIA) |
| Logarithmic Mean Divisia Index (LMDI) method for crop water footprint assessment |
| Lorenz curve and Gini index to calculate household income distribution under Land Use Consolidation (LUC) programme |
| Mathematical models for estimating Volatile Solids (VS) and biodegradable (dVS) outputs of lactating dairy cows |
| Matrix factorisation technique - Simplex Volume Maximisation (SiVM) |
| Meal coding system |
| Mermaid - shellfish sanitation model |
| Method for linking longitudinal retail price data with objective, nutrient-based ratings of the nutritional quality of foods and beverages |
| Methodology for estimating the benefits of vitamin A (VA)-related interventions |
| Minimal data foot printing solution using mixed modelling procedure |
| Minimod - micronutrient intervention planning model |
| Model for estimating nutrient addition contents to staple foods fortified simultaneously |
| Monitoring Results for Equity System (UNICEF) |
| Multi-indicator sustainability assessment |
| Multi-region input–output (MRIO) framework |
| Multiple Indicator Multiple Cause (MIMIC) models |
| National accounts data to estimate fixed capital in agriculture |
| National Water, Food & Trade (NWFT) modelling framework |
| Neural Networks (NN and ANN) |
| New methods for including gender-specific information in agricultural risk assessments |
| New model for estimating dietary intake of nutrients and additives in animal products |
| Nutrient production analysis: global agricultural and nutrient production by farm size |
| Nutrition footprint |
| NUTRitional Postharvest Loss (NUTRI-P-LOSS) methodology |
| On-farm biodiversity model (model to predict butterfly and plant species richness on field margins) |
| One health |
| Optimization modelling for selection of national micronutrient interventions |
| Other network/DAG models |
| Political Economy Analysis (PEA) |
| Polluted basin recovery planning water quality classification and mathematical integrity model |
| PolyCrop (PC) hydrologically based, multi-year daily crop model |
| Predictive model for the level of pathogenic bacteria in raw dairy |
| Preventable Risk Integrated ModEl (PRIME) comparative risk assessment model |
| Probabilistic model to evaluate Food-Based dietary Recommendations (FBR) |
| Random forest model |
| Rapid Agricultural supply chain Risk assessment (RapAgRisk) |
| Real-value Genetic Algorithm Support Vector Regression (RGA-SVR) |
| ReCiPe method for the Life Cycle Impact Assessment (LCIA) |
| Recursive-Dynamic Computable General Equilibrium (DCGE) model |
| Rule-based analysis for farm management |
| Scenario simulation based Stocks and Flows Framework and economic cost of disease burden projections |
| Simulation integrating seasonal forecasts into an ongoing pilot insurance scheme for smallholder farmers |
| simulation model combining deterministic and probabilistic approaches to estimate habitual iodine intake |
| Simulation of the anthropised water cycle combining a hydrological model and a decision support system |
| Soil and Water Assessment Tool (SWAT) |
| SPARE:WATER |
| Spatial autocorrelation analysis and a Geographically Weighted Regression (GWR) model |
| SPAtially Referenced Regressions On Watershed attributes (SPARROW) model |
| Spatially-explicit water balance model (CWUModel) |
| SSM-legumes, a crop model generic to legume species |
| Stochastic Frontier Approach (SFA) to Food Environments and BMI |
| STochastic Impacts by Regression on Population, Affluence and. Technology (STIRPAT) Model |
| Support Vector Regression (SVR) model |
| Sustainable Nutrition Security (SNS) model |
| Swiss Agricultural Life Cycle Assessment for Soil Quality (SALCA-SQ) |
| Targeting AGricultural water Management Interventions (TAGMI) |
| Time series analysis and Theil-Sen estimator of slope for irrigation assessment |
| Unconditional Quantile Regression estimator |
| Virtual water trade/footprints |
| Volumetric Water Footprint and stress-weighted WF |
| VOR and VORS model - Adapted: Vigor (V) [Net Primary Production (NPP), O (Organization: area proportion of nature ecosystem, Shannon Diversity Index (SHDI), Contagion Index (CONTAG), R (Resilience: ecology elasticity), and S (Service: water conservation, soil conservation)] |
| Water footprint assessment (WFN, post-2010) |
|  |
|  |

**Table S2‑10 List of unique research design methods by domain**

| **RESEARCH DESIGN** |
| --- |
| 'CSI Pollen' citizen science sampling |
| 'Total Diet Study Exposure' (TDS-Exposure) |
| Estimating global burden of Food Borne Diseases (FBDs): structured expert elicitation |
| Estimation of relative bias between survey design choices: self-reporting, head of household and proxy response bias (Alternative respondent criteria) |
| Estimation tools for extended harvest production measurement |
| Household measures approach to reporting portion size |
| Methods for including gender-specific information in agricultural risk assessments |
| Point-source testing of pathogens in public water supply |
|  |
|  |
